# How Source Attribution Visualization Shapes User Attention and Preference: An Eye-Tracking Study of Four AI Chatbot Layouts

**DOI:** 10.3390/jemr19040089

**Published:** 2026-08-14

**Authors:** Junho Cho, Dokshin Lim

**Affiliations:** 1 Department of Visual Communication Design, School of Design, Hongik University, 94 Wausan-ro, Mapo-gu, Seoul 04066, Republic of Korea; planning523@gmail.com; 2 Department of Mechanical and System Design Engineering, Hongik University, 94 Wausan-ro, Mapo-gu, Seoul 04066, Republic of Korea

**Keywords:** AI chatbot, source attribution, eye tracking, visual attention, perceived trustworthiness, generative AI, human–AI interaction, visualization

## Abstract

As generative AI chatbots become a primary information channel, users increasingly accept answers without verification, and citations can raise trust even when sources are irrelevant or fabricated. How source-attribution visualization shapes the visual preconditions of verification remains unknown: users can notice, read, or compare a source without clicking. This within-subjects eye-tracking study (*N* = 23; 92 trials) evaluated four attribution visualizations abstracted from commercial AI chatbots and rendered as simulated screens: inline component (sentence-end chips), card list (cards above the answer), side panel (adjacent panel), and raw hyperlink (bare URLs), combining gaze metrics, surveys, and interviews. Repeated-measures ANOVAs revealed strong layout effects on source discoverability and engagement, largely robust to sensitivity checks (the panel’s discovery latency was order-sensitive): the card list was discovered almost immediately, with the raw hyperlink last. Yet no self-reported measure differed detectably. The most frequently nominated format, the inline component, attracted about half the dwell time of the stand-alone formats, whose prolonged fixations suggested citation-to-text mapping cost rather than genuine engagement. This attention–preference gap means both must be measured jointly. We contribute a four-layout gaze-based comparison, a reproducible participant-level analysis workflow, and three design principles (pre-click identifiability, sentence-level claim–source mapping, and in situ preview) within a proposed two-stage attribution architecture.

## 1. Introduction

### 1.1. Background

The rapid popularization of generative AI has produced a distinct shift in information-seeking behaviour, from the traditional cross-verification style of web search toward the uncritical acceptance of AI-generated answers, a pattern of verification-free information consumption [1,2]. In a recent South Korean press survey, 86% of university students reported using AI in their studies [3]. This pattern is not confined to South Korea. In the United States, 45% of the population aged 18 to 64 uses generative AI, an adoption curve faster than that of the personal computer or the internet [4]. Across 45 markets, 10% of respondents now use AI chatbots for news weekly, with use in South Korea doubling year on year, yet only 4% always or often click through from a chatbot answer to the original source, versus 19% from search engines, which corroborates verification-free consumption at a global scale [2]. In this environment, AI chatbots are no longer mere conversational agents; for many users, they function as the most trusted single source of information, even though large language models are known to produce fluent but unfaithful or fabricated content [5].

This shift is consequential at the interface level because generative AI systems increasingly replace ranked lists of documents with a synthesized answer. This compression is useful, but it also relocates evidentiary work. In conventional search, users can compare result snippets, publishers, and URLs before choosing a document; in an AI answer, claims arrive as fluent prose, and sources appear as secondary interface elements: citation chips (compact labelled markers attached to individual sentences), cards (thumbnail summaries showing a source title and outlet), side panels (separate columns of source previews beside the answer), footnotes (numbered notes collected beneath the text), or links (bare URLs). These formats are not hypothetical abstractions: a pre-study survey of six widely used commercial services found all four of the layout types examined in this study deployed in practice frequently combined within a single answer (Section 2.2). The source interface, therefore, does more than display metadata. It determines when evidence becomes visible, how easily a claim can be connected to that evidence, and how much interaction is required to inspect the original material. Because the same underlying answer can be presented with structurally different attribution formats, the structural form of the source attribution user interface (UI) may determine whether citations invite genuine verification or merely simulate credibility [6,7]. Understanding this mechanism requires moving beyond self-reported trust toward direct observation of users’ visual behaviour at the moment they encounter source cues.

### 1.2. Citations as Trust Cues

A substantial body of credibility research has argued that interface cues trigger heuristic credibility judgments before users fully inspect content. The MAIN model [8] proposes that modality, agency, interactivity, and navigability affordances convey cues that invoke cognitive heuristics about content quality (see also [9]), while prominence–interpretation theory [10] holds that an element can affect credibility assessment only if it is first noticed (prominence) and then evaluated (interpretation). Consistent with these frameworks, users rarely engage in effortful verification and instead rely on readily available surface cues (source information, presentation quality, and content plausibility) under time constraints when judging online information [11,12,13]. In algorithmic systems, transparency can improve trust after an expectation violation, but excessive information can reduce trust or increase processing demands [14]. Human–AI interaction guidelines, therefore, recommend making system capabilities and uncertainty legible without overwhelming users [15].

The appropriate design target is calibrated reliance rather than maximal trust. Explanations and transparency cues can produce overreliance when they are interpreted as evidence of competence rather than as material to inspect [16]; cognitive-forcing interventions can reduce uncritical acceptance by requiring users to engage with evidence before acting [17], and interface-level cues have likewise been shown to curb overreliance on answers from large language model (LLM)-based search [18]. Social and provenance-oriented transparency likewise argues that explanations should answer practical questions about who or what supports a system output and in what context [19,20]. Source interfaces in generative AI occupy this same design space: they can either support verification or function as a persuasive badge [21].

Recent empirical work has documented how powerfully the citation heuristic operates in AI answer systems. Ding et al. [6] showed that attaching citations to LLM-generated answers significantly increased user trust even when the citations were randomly selected and irrelevant to the answer content, while the act of checking citations was associated with lower self-reported trust. In a large-scale experiment, Li and Aral [22] similarly found that reference links and citations significantly raised trust in generative AI search even when the underlying information was incorrect or fictitious. Direct audits of generative search engines report incomplete citation coverage and citations that do not entail the statements they accompany [7,23], and field-oriented analyses of AI answer engines reinforce the concern: participants hovered over an average of only two sources per session, and one participant reported checking only that sources exist, without any verification of their content [1]. Trust in AI answer platforms is further conditioned by platform-level cues, differing from trust in conventional search and encyclopaedic sources [24], and LLM-powered search can reshape the diversity of information seeking itself [25]. Together, these findings indicate that users form trust from the mere presence of source cues rather than from the verifiability of the cited material: citation presence and citation verifiability are not equivalent.

However, prior studies share a common methodological limitation: they rely predominantly on behavioural outcome data such as post hoc trust ratings or click logs. Such data reveal that users “felt” trust or did or did not click a source, but they cannot explain what visual search strategies unfolded at the moment users perceived the source UI: which attribution formats were discovered quickly, which were scrutinized deeply, and which were skipped entirely. This process-level gap motivates the present eye-tracking approach.

### 1.3. Reference Presentation in Conversational Search

Source interfaces in current conversational systems vary along several dimensions: whether references are collocated with claims or separated into a list; whether provenance is semantically labelled or represented as a raw URL; whether details are persistent or revealed on demand; and whether interaction produces bidirectional highlighting. Ouyang and Narechania [26] documented substantial variation in the presentation, quantity, and quality of references across nine LLM-based conversational systems and observed that users rarely interact with references. He and Liu [27] experimentally compared four presentation designs and showed that transparency is not a unitary property: layout changed attention, interaction, and persuasion. Theirs was a crowdsourced between-subjects experiment in which attention was indexed by hover and click logs rather than gaze.

These findings motivate a finer-grained visual account. Clicks and hovers are important downstream behaviours, but the absence of a click does not imply the absence of examination. Search research has long shown that non-click interaction traces can reveal evaluation behaviour that click logs miss [28]. Eye tracking adds direct temporal measures of discovery and visual allocation: it can reveal whether an apparently unused source component was never noticed, briefly checked, or repeatedly revisited while the user compared it with the answer. However, gaze is not self-interpreting. A long dwell may indicate interest and careful reading, but it may also indicate confusion, dense text, or unsuccessful search. We therefore avoid treating more gaze as inherently better and use interview evidence to distinguish productive inspection from mapping cost. This interpretive stance is central to our analysis.

### 1.4. Eye-Tracking Metrics and Area of Interest Validity

Eye tracking provides a process-level lens on information evaluation grounded in the eye–mind hypothesis, which holds that what a person is looking at indicates what they are currently perceiving, attending to, or processing [29]. Time to first fixation (TTFF) indexes the discoverability of an interface element and the efficiency of attentional guidance [30,31], whereas fixation duration and fixation count characterize the later allocation of visual processing devoted to it [31,32]. Visit and revisit measures complement these indices. The number of visits to an area of interest, and the frequency of returns to it, indicate whether an element is consulted once or repeatedly reinspected during comparison with other content [31,32]. Beyond absolute durations, usability research has long expressed attention to an element relative to total viewing time: the proportion of fixation time on an area of interest dates back to the instrument-scanning studies of Fitts et al. [33]; Jacob and Karn [34] catalogued it as one of the most frequently reported eye-tracking metrics in usability studies. Such proportional dwell measures have been used to quantify how attention is distributed across the components of search results [35,36], web pages [37,38], and choice environments [39] and remain central in recent applied eye tracking, from mobile augmented-reality usability [40] to the allocation of attention between generative AI answers and conventional results on search pages [41]; systematic reviews likewise list area of interest (AOI)-level dwell and proportion metrics as standard practice [42,43]. Reading of web content typically follows an F-shaped scanning pattern anchored to the upper-left region of the page [44], implying that the spatial placement of source cues (inline within the reading flow vs. separated into peripheral regions) should systematically alter their discoverability. In addition, pupil diameter provides a covert physiological index of cognitive load during information processing [45,46], enabling an assessment of whether particular attribution formats impose greater mental effort.

These measures are useful only when the AOI definition and unit of analysis match the research question. AOI size, location, and boundaries influence hit classification, and poorly specified AOIs can threaten both reliability and construct validity [47,48]. Recent methodological guidance stresses transparent AOI construction, calibration quality, missing-data handling, and the distinction between gaze samples and derived events [49,50]. AOI size is a particularly consequential researcher degree of freedom: the chosen AOI extent alters measured attention and can change statistical conclusions [47,51], dwell-based measures scale strongly with element size on web pages [37], and recent work either controls for AOI size statistically or replaces areas with point-based estimators [52]. When regions of unequal size must be compared, methodological guides recommend normalizing the measured value by AOI area for graphical stimuli or by the amount of content for textual stimuli ([53]; see also [54]). Our study raises a specific validity problem: each interface contains a different number of source rectangles. If rectangle-level metrics are pooled as though each were an independent participant observation, conditions with more rectangles obtain larger apparent sample sizes, and multiple values from the same participant are treated as independent, a form of pseudoreplication. Recent eye-tracking work on AI-mediated content underscores how little is known about attention to attribution elements specifically: AI-use disclosure labels change readers’ gaze behaviour, yet AOI-level isolation of the disclosure region has been explicitly deferred to future work [55], and eye-tracking studies of generative answer pages have so far excluded in-text reference links from their stimuli altogether [41]. Despite the maturity of eye-tracking methods in usability research, to our knowledge, no study has systematically compared the four major source-attribution UI typologies observed in commercial AI chatbots using gaze-based process measures while preserving the participant-by-condition experimental unit; the present study addresses both gaps.

### 1.5. Research Objectives and Hypotheses

The present study aims to analyse the relationship between actual gaze patterns, measured via eye tracking, and subjective trustworthiness. The practical objective is to identify the attribution UI model that best supports the early, measurable stages of verification (noticing and attending to sources) while sustaining an appropriate sense of trust, thereby informing a trust-centred UI direction for AI services. Four representative typologies were abstracted from commercial AI answer systems: (A) an inline component (chip) type attaching a compact source chip at the end of each supported sentence; (B) a card list type presenting sources as horizontally arranged cards above the answer; (C) a side panel type aggregating source previews in a separate right-hand panel; and (D) a raw hyperlink type listing bare URLs beneath each answer section. Based on the literature reviewed above, the following hypotheses were proposed:

**H1.**  *Stand-alone source UI variants with high visual independence (card list and panel types) will be detected more rapidly than UI variants embedded within or listed beneath the answer text (component and hyperlink types). This expectation follows from the salience of visually bounded, stand-alone modules: element size and salience are strong predictors of gaze allocation on web pages [37], placement relative to the F-shaped entry zone governs how early content is reached [44], and recent experimental work shows that the presentation format of references alone redirects attention in conversational search [27].*

**H2.**  *Stand-alone source UI variants, in which source information is organized into a single semantic chunk, will elicit greater visual attention and longer total duration of fixations (TDF) than non-chunked UI variants (inline chips and section-end links). Chunked presentation is expected to sustain processing once discovered: gaze concentrates within coherently grouped regions [32,56], and recent eye tracking of search pages shows aggregated generative-answer modules attracting substantial dwell [41].*

**H3.**  *The hyperlink-type source UI, which adopts a familiar web-environment format, will (H3a) induce lower cognitive load and (H3b) engender higher perceived trustworthiness than the other UI typologies. (Cognitive load is indexed by exploratory pupillometry; Section 3.6.) Familiar interface conventions invoke credibility heuristics that reduce evaluative effort [8], platform-familiarity cues continue to condition trust in present-day AI answer systems [24], and dense or unfamiliar information can raise processing demands and depress trust [14]. Pupil diameter supplies the corresponding physiological index of load, from the classical demonstrations [45,46] to contemporary reviews [57,58].*

**H4.**  *The promptness of initial detection (TTFF) of a source UI will be dissociable from both the gaze fixation duration devoted to actual information verification and the subsequent formation of user trust. Prominence and interpretation are theoretically separable stages of credibility assessment [10], and recent experiments show that citations can raise trust without any inspection of their content [6,22], while AI-search users verify almost nothing in practice [1], so early detection need not translate into scrutiny or trust.*

**Figure 1:**
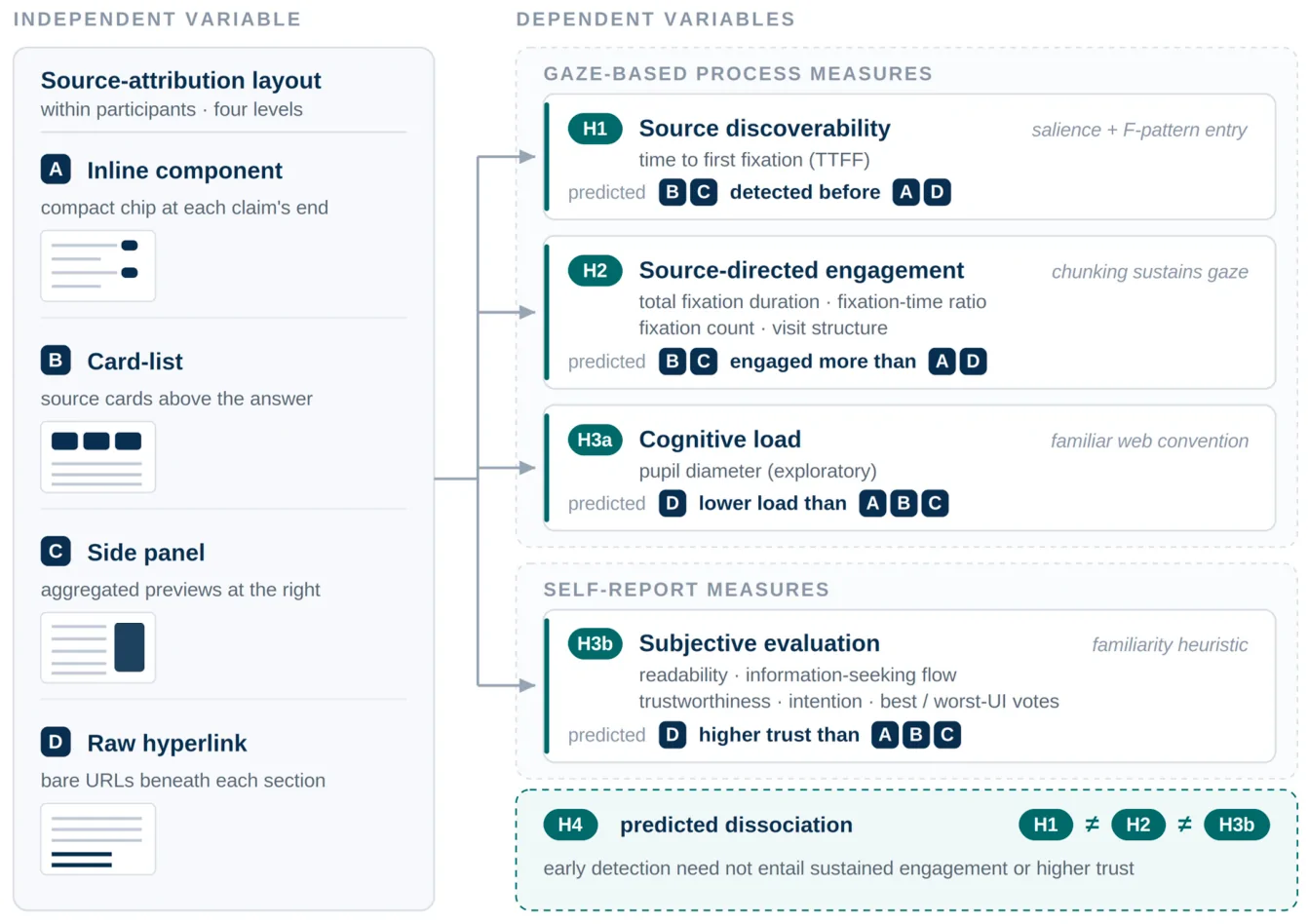
Conceptual framework of the study. The within-participant independent variable (source-attribution layout, A–D) is hypothesized to shape source discoverability (H1), source-directed engagement (H2), cognitive load (H3a), and subjective evaluation, including perceived trustworthiness (H3b). Navy marks the layouts; teal marks the hypotheses and outcome families. Colour identifies these two roles and encodes no ranking or evaluation. Letter chips restate the contrast each hypothesis predicts between groups of layouts, and no ordering is predicted within a group. Italic notes give the theoretical grounds for each prediction. The strip at the foot states H4, which predicts that discoverability, engagement, and trust need not co-vary.

Figure 1 summarizes the hypothesized relationships among the four layouts and the outcome measures.

In addition to these hypotheses, the study qualitatively explores which structural properties of attribution UIs users experience as supporting, or obstructing, their verification of AI-generated answers. A further methodological objective concerns measurement validity: the four layouts contain different numbers and sizes of source areas of interest (AOIs), and treating each AOI rectangle as an independent observation would inflate the sample for layouts with more source elements and violate the repeated-measures unit. We therefore develop and report a documented, reproducible participant-level reconstruction pipeline for Tobii exports.

Specifically, this paper makes four intended contributions. First, it reports an eye-tracking comparison of four source-attribution layouts that separate source discoverability from source-directed engagement. Second, it documents a reproducible participant-level reconstruction pipeline for Tobii exports in repeated-measures designs with unequal numbers of source AOIs. Third, it provides evidence that rapid discovery, prolonged attention, and perceived trust do not collapse into one ranking of interface quality. Fourth, it proposes a verification-oriented design framework (Notice, Attend, Map, Act) together with three design principles embodied in a two-stage attribution architecture combining rapid source cues, explicit claim–source mapping, and progressive disclosure, offered as an empirically testable design proposal.

## 2. Materials and Methods

**Figure 2:**
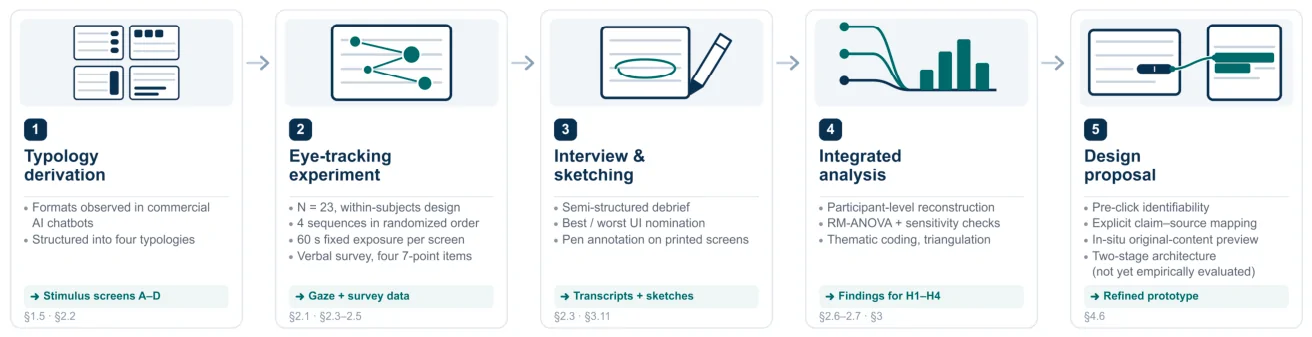
Overview of the research process: source-attribution formats observed in commercial AI chatbots were structured into four stimulus typologies (A–D), evaluated in a within-subjects eye-tracking experiment with post-stimulus surveys, followed by a debrief interview with sketching; an integrated analysis of gaze, survey, and interview data; and a verification-oriented design proposal.

Figure 2 provides an end-to-end overview of the research process; the following subsections detail each stage.

### 2.1. Participants and Ethics

In total, 24 adult participants (of a planned 30) with prior experience using generative AI chatbots were recruited through convenience sampling from a university population. One participant whose recording fell below the pre-specified gaze-quality criterion (valid gaze sample rate ≥ 70%) in at least one condition was excluded. Because the repeated-measures analysis required complete four-condition data, all four trials from that participant were removed, yielding a final sample of 23 participants (18 women, 5 men; all university students in their twenties) and 92 trials. Participants were aged 20–28 years (born 1998–2006). Of these, 16 regularly used three or more AI services; 13 majored in design and 10 in non-design fields. Data were collected in individual laboratory sessions between 3 and 23 June 2026. A sensitivity calculation contextualizes this sample: with *N* = 23, four within-participant levels, α = 0.05, and power = 0.80, the design detects effects of f ≈ 0.27–0.31 (ηp^2^ ≈ 0.07–0.09) under conventional assumptions (ρ = 0.30–0.50, ε = 0.85); smaller effects cannot be ruled out. Recruitment was additionally informed by the heatmap stability curve reported by Pernice and Nielsen [59] (p. 58), whereby approximately 20 users recover the dominant gaze distribution (R^2^ ≈ 0.70). (This criterion addresses the stability of aggregate gaze distributions, not inferential power for the ANOVA or survey comparisons, which is covered by the sensitivity calculation above.) Comparable eye-tracking studies have derived robust behavioural insights from samples of 10–20 participants [60,61,62].

All participants were regular users of generative AI chatbots: 17 reported using an AI chatbot almost daily, 4 used one two to three times per week, and 2 used one approximately weekly. ChatGPT was the most widely experienced platform, followed by Gemini, Claude, Perplexity, NotebookLM, Genspark, and Grok (multiple responses permitted). Participants had normal or corrected-to-normal vision, and conditions incompatible with eye tracking (e.g., hard contact lenses or decorative lenses) constituted exclusion criteria [59].

The study was conducted in accordance with the Declaration of Helsinki and approved by the Institutional Review Board of Hongik University (protocol code 7002340-202606-HR-003, 2 June 2026). All participants received an explanation of the study and provided written informed consent prior to participation. The task involved reading simulated AI answers and did not expose participants to deceptive source-quality manipulations.

### 2.2. Experimental Design and Source-Attribution Layouts

**Figure 3:**
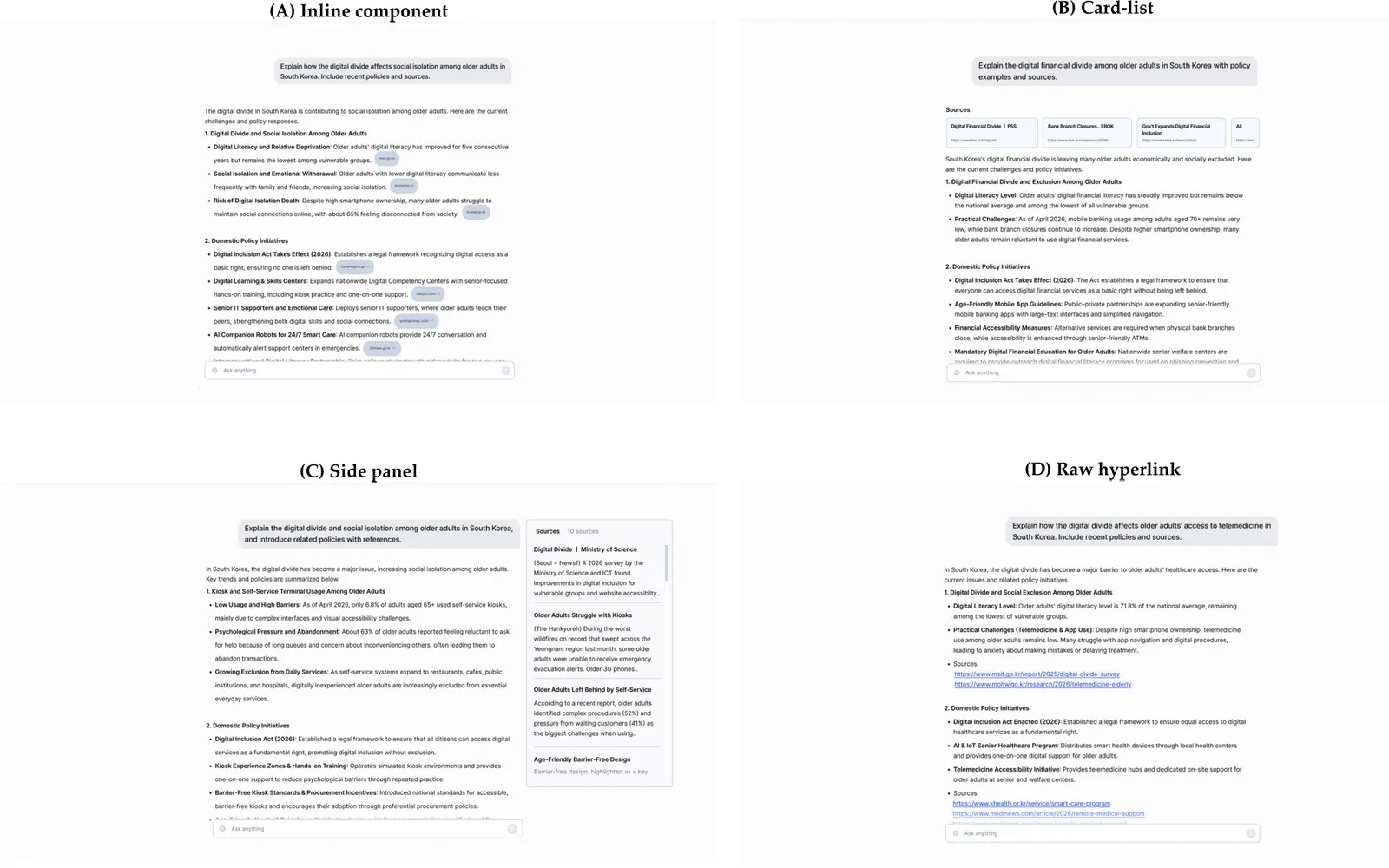
Four source-attribution layouts used in the study: ((**A**) inline component; (**B**) card list; (**C**) side panel; and (**D**) raw hyperlink). English translations of the four screens are shown for readability; participants viewed the original Korean-language versions, which had identical layouts, source placements, and fold position.

The study employed a within-subjects comparative design with four experimentally constructed Korean-language AI chatbot answer screens, each implementing one source attribution UI typology (Stimuli A–D; Figure 3). No commercial chatbot platform was used. The four screens were custom-built simulated answer pages whose visual shell followed the common layout conventions of commercial AI chatbots, so that no platform branding or platform-specific behaviour could confound the comparison. To isolate the effect of attribution format, the conversational scenario, answer structure, information density, visual style of the chatbot shell, and overall length were held constant across conditions; only the placement, pre-click information content, and body-text mapping of the source cues were manipulated. One geometric exception was unavoidable: the side panel condition reflowed the answer column to a narrower width, an intrinsic property of the layout being tested; this reflow is a plausible partial contributor to the panel’s longer dwell, slower discovery, and larger pupil and is revisited in Section 3.6 and Appendix C. The four answer screens addressed four parallel subtopics of a single social issue (the digital divide among older adults in Korea: social isolation, financial-service exclusion, kiosk and unmanned-terminal use, and telemedicine accessibility), so that no condition benefited from intrinsically more engaging content. The subtopics were assigned as follows: A, social isolation; B, exclusion from financial services; C, kiosk and unmanned-terminal use; and D, access to telemedicine. This pairing maximized ecological variety but means that content and layout were not fully crossed; we return to this point in Section 5. Table 1 summarizes the manipulations; Table 2 reports the source-region geometry of each condition.

The layouts intentionally differed in source-region size, number of elements, density, and location because these properties constitute the design treatment. We therefore treat raw source-directed attention as the primary outcome; a simple area-normalized density is reported only as a secondary descriptive lens, not as a corrected or “truer” measure of interface effectiveness (Section 2.5). The unit of comparison is therefore each layout as deployed in commercial practice (a package of format, source count, source-region area, and column geometry) rather than an isolated formal factor. Each format abstracts a recognizable presentation method: claim-adjacent citation chips, horizontally arranged source cards, aggregated source previews in a side panel, and bare URL links appended to answer sections. A pre-study survey of widely used commercial services conducted in March 2026 (ChatGPT, Gemini, Claude, Perplexity, Copilot, and the Korean service Wrtn) informed this abstraction: all four methods were observed in commercial deployment, frequently in combination within a single answer. The same service, however, presented sources differently across model versions and subscription tiers and could differ between sessions even for an identical prompt; implementations have also continued to change since the survey: by July 2026, Gemini, which at the time of the survey only annotated the provenance of its answers, had introduced per-claim source chips, and DeepSeek, a widely used service not included in the survey, combined inline numbered markers with an aggregated side panel within a single answer. Parallel recombinations were observed in four further services in the same month. Kimi paired claim-adjacent chips, naming the cited outlet with an aggregated reference panel opened on demand. Grok likewise attached outlet-named chips to individual claims, adding a hover card that previews the cited article’s title and line-numbered fragments of the page text, together with an on-demand panel disclosing the search queries and pages it consulted. Meta AI marked individual claims with icon-only anchors that disclose only the cited page’s title and linked domain on hover (for portal-syndicated articles, the portal’s domain rather than the original outlet’s), alongside a similar on-demand source list. Vibe (Mistral AI) appended labelled, dated source links beneath each claim. Published audits document the same cross-system variability [26]. The four layouts, therefore, abstract the recurring presentation methods themselves, rather than the practice of any particular product at any particular time.

### 2.3. Procedure

The session comprised three phases. In the preparation phase, participants provided informed consent and were introduced to the experimental task. A calibration and validation procedure was administered using Tobii Pro Lab, and recordings displaying warning indicators or off-centre pupil capture were recalibrated before proceeding.

In the execution phase, participants completed four eye-tracking–survey sequences, one per stimulus, in randomized order. Each sequence consisted of (i) a task interstitial page introducing the upcoming topic, read at the participant’s own pace; (ii) the AI chatbot answer screen containing the manipulated source attribution UI, presented for a fixed duration of one minute; and (iii) a verbally administered post-stimulus survey in which participants responded aloud to four seven-point Likert items about the screen they had just viewed. After the fourth sequence, a semi-structured debrief interview was conducted; it concluded with a brief sketching exercise in which participants annotated printed copies of the four screens with a pen, marking elements they would change and explaining what made an interface better or worse. (These annotations were thematically coded into Table 9).

The fixed 60 s exposure guaranteed equal opportunity of exposure to all AOIs and maximized the comparability of cumulative fixation data across participants [49,59]. Presentation order followed a per-participant random sequence list generated in advance (each sequence an independent shuffle of A–D via spreadsheet SORTBY/RANDARRAY, with no balance constraint), prepared for a planned sample of 30; because the list was fixed before data collection, no adaptive rebalancing occurred when recruitment closed early. The resulting allocation was not balanced: condition C appeared first for 11 participants, whereas A, B, and D each appeared first for four (Appendix B). Such an allocation is improbable under uniform per-participant randomization (binomial P(X ≥ 11) ≈ 0.015, or ≈ 0.058 after accounting for the post hoc selection of the largest of the four condition counts); it is flagged transparently here and addressed through the sensitivity analyses reported in Section 2.7 and Section 3.9.

In the evaluation phase, a semi-structured post-interview was conducted: (Q1) whether the manner of source attribution influenced their trust; (Q2) which UI they regarded as the best, with freely described and sketched improvement ideas; (Q3) which UI they regarded as the worst, likewise with sketched improvements; and (Q4) any further improvement suggestions for the remaining UIs. Sketching on paper was encouraged to elicit concrete design-level feedback.

Participants were instructed to imagine that they were exploring candidate topics for a final presentation in a liberal arts course using an AI chatbot. They would examine four candidate topics, one per screen, and ultimately had to select the topic whose answer they judged to be supported by the most solid evidence. For each screen, they were asked to read the AI answer freely while judging whether the information was sufficiently trustworthy and suitable for citation as academic material. This framing was designed to induce an evidence-oriented reading goal, making source cues task relevant without directing attention explicitly to any interface element.

All four screens presented an identical prompt template (“Please describe the current situation of [subtopic] among older adults caused by the digital divide recently emerging in Korean society, together with domestic policy cases addressing it, with sources.”) and a structurally parallel two-part answer (current situation; domestic policy cases). Stimulus A embedded compact source chips (e.g., abbreviated domain labels) at the end of each supported statement. Stimulus B displayed three source cards (title + URL) plus a “show all” control above the answer. Stimulus C aggregated source previews (title + body excerpt) in a right-hand panel labelled with the number of sources (the header announced 10 sources, of which four preview entries were visible without scrolling, corresponding to the four panel AOIs in Table 2). Stimulus D listed bare URLs beneath each answer section. In all four stimuli, the answer column extended beneath a fixed input field anchored at the same vertical position at the bottom of the frame, so the final line of body text was partially occluded identically across conditions, reproducing the fold of commercial chat interfaces without differential information loss.

### 2.4. Apparatus, Setting, and Data Quality

**Figure 4:**
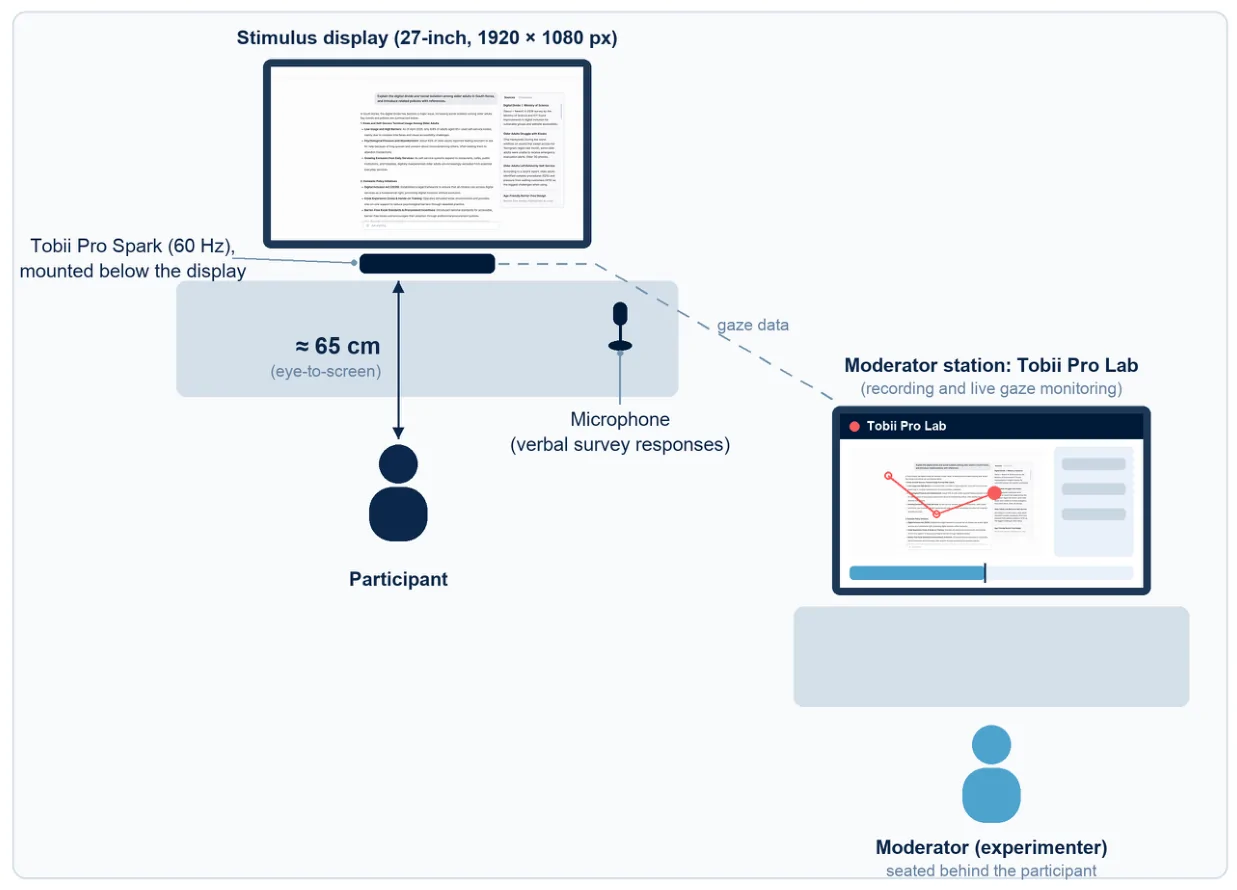
Schematic top view of the experimental setup. The participant viewed each stimulus on a 27-inch display (1920 × 1080 px) at approximately 65 cm eye-to-screen distance, with the Tobii Pro Spark eye tracker (60 Hz) mounted below the display. A desktop microphone recorded the verbal post-stimulus survey responses (Section 2.3). The moderator recorded and monitored gaze data in Tobii Pro Lab at a separate station behind the participant. The software view in the figure is a schematic representation.

The experiment was conducted in a controlled laboratory environment using a Tobii Pro Spark screen-based eye tracker. This single-camera binocular device, which utilizes both bright- and dark-pupil tracking, operated at a sampling frequency of 60 Hz [63]. Stimuli were displayed on a 27-inch monitor (16:9 aspect ratio; 1920 × 1080 px), with participants seated at approximately 65 cm, within the device’s optimal operating range (45–95 cm) and head-movement tolerance area (35 × 35 cm) [63]. Figure 4 shows the experimental arrangement. The manufacturer specifies system accuracy of 0.45° and precision of 0.26° RMS under optimal conditions [63]; observed accuracy in the recorded data is reported in Section 3.1, following the recommendation to determine data quality empirically for each recording rather than to rely on manufacturer specifications [64].

Data were recorded and processed in Tobii Pro Lab (v. 25.23) using the Tobii I-VT fixation filter (velocity threshold 30°/s computed over a 20 ms window; moving-median noise reduction over three samples; averaged binocular gaze; gap fill-in interpolation disabled; adjacent fixations merged when separated by less than 75 ms and 0.5°; fixations shorter than 60 ms discarded; pupil diameter processed with Tobii’s noise-reduction filter), with calibration and validation performed at the start of each session and recalibration integrated into the workflow as needed.

Across the 92 included trials, the proportion of samples with at least one valid eye ranged from 84.7% to 99.8% (M = 95.0%, SD = 2.72); mean calibration accuracy in the export was 1.20°, and mean validation accuracy was 0.99°. Observed accuracy was thus coarser than the manufacturer’s optimal-condition specification (0.45°); because the smallest chip-level AOIs (≈5200 px^2^) are of comparable scale to a 1.2° error radius, the primary analyses rely on the merged-source AOI, which reduces, though does not eliminate, boundary misclassification risk for the inline component condition. A dilation sensitivity analysis, re-classifying all 19,102 fixations with every AOI boundary expanded by 0.5° and by 1.0°, left the discoverability results unchanged at every level (the same three Holm-significant TTFF contrasts), indicating that hit-classification noise does not drive the primary discovery findings. Engagement metrics for the inline chips grew with dilation, as expected geometrically: expanded chip boundaries increasingly absorb fixations on the immediately surrounding body text. The original tight boundaries are therefore retained as the valid operationalization of source-directed attention.

Source AOIs were defined for every source-bearing region of each stimulus: seven rectangles for the component type (one per inline chip cluster), four for the card list type (three cards and the “show all” control), four for the panel type, and two for the hyperlink type (Table 2). Because a raw AOI-level comparison would confound condition effects with the number and size of constituent rectangles, all source AOIs within a condition were merged into a single-source AOI per stimulus, and all ocular metrics were aggregated to one value per participant per condition prior to inferential analysis (Section 2.6). The 60 s exposure of the answer screen defined the time of interest (TOI) for all analyses.

### 2.5. Measures

This study adopts the eye–mind hypothesis [29] as its methodological premise: gaze location indexes the current focus of perceptual and cognitive processing. Four primary ocular outcomes were computed on the merged-source AOI of each participant-condition trial (Table 3). Time to first fixation (TTFF) measured the latency from stimulus onset to the first fixation within the source AOI, indexing discoverability [30]. Total duration of fixations (TDF; referred to informally as dwell) measured cumulative fixation time within the source AOI, indexing sustained source-directed processing [31,32].

The source fixation-time ratio expressed source-AOI fixation time as a percentage of the participant’s total fixation duration in the trial, indexing relative attention allocation [33,34,35]; this denominator (rather than the fixed 60 s display interval) excludes saccades, blinks, and tracking loss from the attention-allocation denominator. Condition means of this denominator were nearly identical (46.6–47.2 s), so the ratio comparisons are not driven by denominator differences. Fixation count captured the number of source-hit fixation events, indexing repeated and sustained processing. All 92 included trials contained at least one source fixation.

Two visit-level descriptors were derived from the same event stream: the number of visits (uninterrupted runs of consecutive fixations within the merged-source AOI, each constituting one entry into the source region) and their mean duration, which together index revisitation proper (Section 3.3).

Two further measures were recorded. First, a secondary area-density metric was calculated as source TDF (in milliseconds) divided by the merged source-region area and scaled to 10,000 px^2^, describing how concentrated fixation time was within the space allocated to sources, following methodological guidance to normalize gaze measures by AOI area when regions of unequal size are compared [53] and earlier per-unit-area attention measures in eye-tracking research [65,66]. Second, average pupil diameter during whole fixations served as an exploratory physiological index of cognitive load [45,46].

The post-stimulus survey operationalized four constructs, each measured with a single seven-point Likert item administered verbally immediately after each stimulus and elaborated with open-ended follow-up (Table 4; full item wording in Table A1, Appendix A). Single items were a deliberate trade-off: they kept the verbal, between-trial protocol brief and uniform, at a reliability cost examined in Section 3.10 and Section 5. The readability item assessed the visual legibility of the screen (font size, line spacing); the information-seeking flow item assessed whether the layout guided the search for key information; the perceived trustworthiness item deliberately targeted the impression of trust conveyed by the screen composition, rather than trust in the content itself, to capture UI-attributable trust under content-controlled conditions; the behavioural intention indicator asked whether participants would want to use the answer in their own assignment or material, treating stated willingness to reuse as a behavioural proxy of trust.

### 2.6. Participant-Level Reconstruction of Tobii Exports

The layouts contained unequal numbers of source rectangles (Table 2), and one fixation could be represented in multiple sample rows or overlap an aggregated AOI and a component rectangle. Directly averaging the per-AOI-item metrics exported by Tobii Pro Lab would therefore answer a different question (when each source item was seen) and could count multiple observations from the same participant as independent.

**Figure 5:**
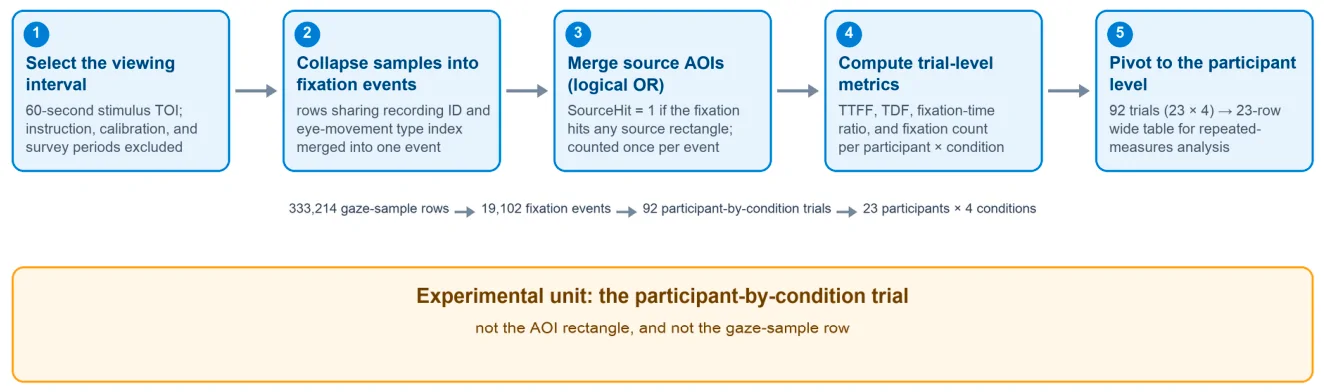
Participant-level reconstruction workflow. The experimental unit is the participant-by-condition trial, not the AOI rectangle or gaze-sample row.

We reconstructed the export in five steps (Figure 5). First, we selected the 60 s answer-viewing interval and excluded instruction, calibration, and survey periods. Second, rows sharing a recording identifier and Tobii eye-movement type index were collapsed into one fixation event. Third, all source AOI-hit indicators within the condition were merged with a logical OR: a fixation hitting one or more source rectangles was coded SourceHit = 1 and counted once. Fourth, one set of trial-level metrics was computed: TTFF = min(start time of fixation j | SourceHit_j = 1); TDF = Σ duration_j for all fixation events with SourceHit_j = 1; fixation-time ratio = source TDF/total fixation duration in the trial. Fifth, the resulting long table contained one row per participant and condition (23 × 4 = 92), and each outcome was pivoted to a 23-row-wide table for repeated-measures inference.

This reconstruction preserves the experimental unit (the participant-by-condition trial, not the AOI rectangle or gaze-sample row) and prevents source-rich layouts from acquiring a larger effective sample merely because they contain more AOI rectangles.

### 2.7. Statistical and Qualitative Analysis

TTFF, TDF, and fixation count were positively skewed, and the fixation-time ratio was bounded. For each outcome, we fit a one-factor repeated-measures ANOVA with layout (A–D) as the within-participant factor, reporting Greenhouse–Geisser-corrected *p* values [67] and partial η^2^ with Cohen’s [68] benchmarks. In the text, F ratios are reported at the corrected degrees of freedom; Table 7 reports the same tests at the nominal degrees of freedom together with the tabulated ε. Because raw-scale ocular metrics violate normality assumptions, we adopted a dual-reporting policy: TTFF is reported on the raw scale for interpretability, while its ln(1 + x) transform enters the primary Holm-adjusted family alongside the other outcomes (Table 7); the engagement outcomes (TDF, fixation count) were analysed on the natural-log scale and the fixation-time ratio, converted from a percentage to a proportion, on the logit scale, with raw-scale means, standard deviations, and distributions reported for interpretation. The offset was applied only to TTFF, for which 10 trials, 8 of them in the card list condition, recorded a first source fixation at 0.00 s, a value for which an unshifted logarithm is undefined; because every trial contained at least one source fixation, these zeros denote immediate discovery rather than missing data. The remaining outcomes contained no zero values and were transformed without an offset. The area-normalized source fixation time was analysed on the raw scale.

To limit multiplicity, the primary omnibus *p* values were Holm-adjusted as one family [69]. Significant outcomes were followed by paired *t* tests on the analysed scale with Holm correction across the six layout pairs; Cohen’s dz characterizes the paired effect size. Figure error bars report within-participant 95% confidence intervals computed with the Cousineau–Morey method [70,71].

We conducted three sensitivity analyses. First, Friedman tests [72] evaluated whether the condition effect remained on the untransformed scale without normality assumptions. Second, participant-fixed models included presentation position (1–4) alongside layout to assess the imperfect order allocation. Third, a leave-out reanalysis repeated the nonparametric tests after excluding the participants for whom the most over-assigned condition appeared first (Section 3.9). The Holm family comprised the four primary gaze outcomes; pupillometry and the area-density metric were exploratory or secondary and are reported outside that family. These models are sensitivity checks rather than a substitute for prospective counterbalancing. Heatmaps accumulated over the full 60 s TOI were generated on the basis of cumulative fixation duration rather than fixation count, as prolonged gaze is a more robust indicator of sustained engagement [32,59].

Survey scores were analysed with repeated-measures ANOVAs (Greenhouse–Geisser-corrected) treating layout as the within-participant factor, matching the participant-level logic applied to the ocular data, and were corroborated by Friedman tests and Holm-adjusted paired contrasts. Exploratory stratified analyses of the gaze and pupillometric data examined three participant variables: AI usage frequency (low: up to 2–3 times per week, *n* = 6, vs. high: almost daily, *n* = 17), platform versatility (single/dual-platform users, *n* = 7, vs. multi-platform users of three or more AI services, *n* = 16), and academic major (design, *n* = 13, vs. non-design, *n* = 10). These analyses are reported descriptively as hypothesis-generating.

Post-interview transcripts and sketches were segmented into meaning units and thematically coded by UI type into layout-specific benefits, verification barriers, and improvement requests, with the number of participants voicing each theme recorded as an index of prevalence within this sample. Coding was performed by the first author and subsequently reviewed by the corresponding author. Because this was a code-then-review procedure rather than independent dual coding, no inter-rater reliability statistic was computed, and the theme counts remain descriptive. The secondary area-density metric is not used as an area-adjusted causal estimate because AOI geometry is an intentional interface feature and dwell time need not increase linearly with area [56,73]; it provides only a complementary description of how concentrated gaze was within the space allocated to sources.

Statistical analyses were computed with custom analysis code, and Claude (Anthropic; Claude Opus 4.8 and Claude Fable 5) assisted with drafting the analysis code and with language editing; key models were additionally cross-checked by the authors in IBM SPSS Statistics (version 30.0.0; IBM Corp., Armonk, NY, USA) and jamovi (version 2.6.26; The jamovi project, Sydney, Australia), with convergent results.

## 3. Results

### 3.1. Data Integrity

**Figure 6:**
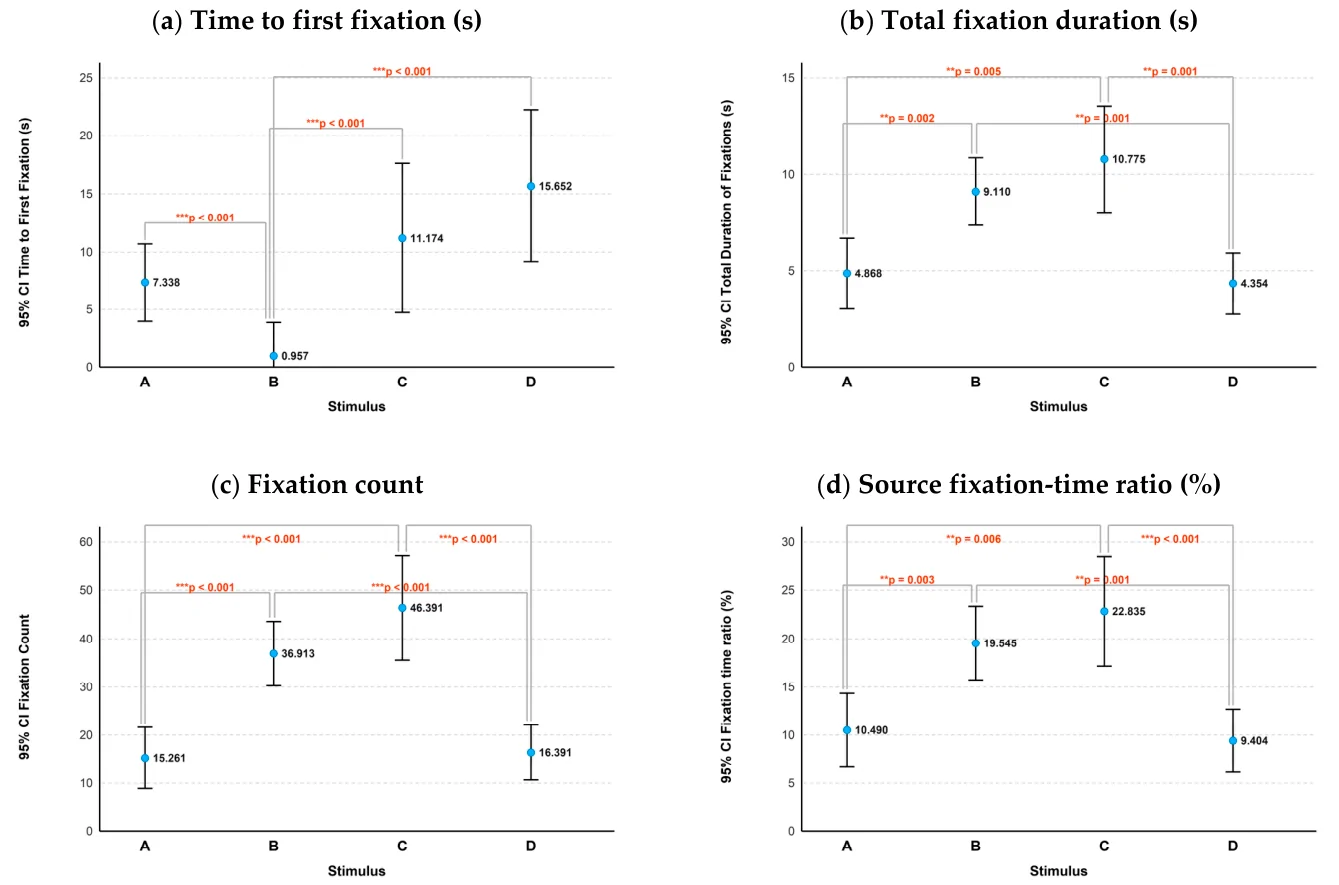
Condition means with 95% confidence intervals for the four primary gaze outcomes across the four source-attribution layouts (A: inline component; B: card list; C: side panel; and D: raw hyperlink): (**a**) time to first fixation (s), (**b**) total fixation duration (s), (**c**) fixation count, and (**d**) source fixation-time ratio (%). Dots are condition means, and whiskers are within-participant 95% confidence intervals (Cousineau–Morey correction); brackets denote significant Holm-adjusted pairwise comparisons from the transformed-scale analyses reported in Section 3.2 and Section 3.3 (** *p* < 0.01, *** *p* < 0.001). Lower confidence limits extending below zero are truncated at zero (durations cannot be negative). The panel’s TTFF in (**a**) is order-sensitive, and the A–C–D contrasts in (**a**) are not significant after Holm correction (Section 3.2 and Section 3.9).

**Figure A1:**
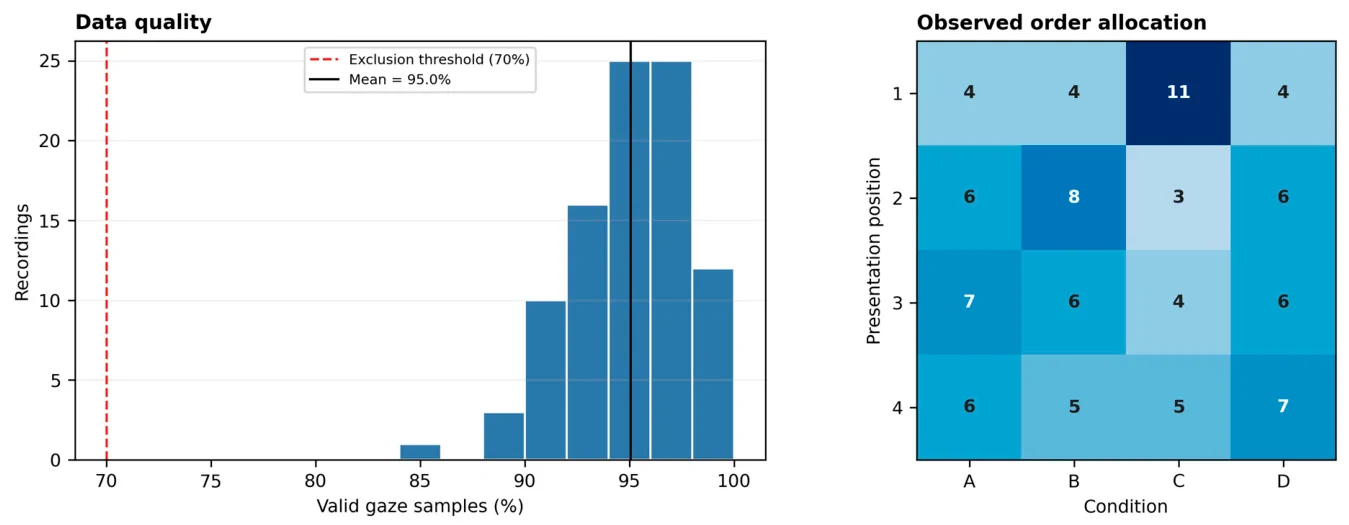
Data-quality distribution and observed condition-by-position allocation. (**Left**): valid gaze sample percentages with the 70% exclusion threshold. (**Right**): heatmap of condition counts at each presentation position.

Recordings were screened against the pre-specified quality criterion of a valid gaze sample rate of at least 70%, the threshold below which excessive blinking or calibration drift compromises the precision of ocular metrics. One participant fell below this threshold and was excluded, leaving 23 participants (92 stimulus recordings) in the final dataset; retained recordings ranged from 84.7% to 99.8% valid samples (M = 95.0%). All retained recordings met the pre-specified gaze-sample quality criterion. The distribution of gaze sample rates and the observed condition-by-position allocation are reported in Appendix B (Figure A1). Table 5 summarizes participant-level gaze outcomes on the raw scale, and Figure 6 shows the corresponding condition means with 95% confidence intervals. The remainder of this section follows the hypotheses. Section 3.2, Section 3.3, Section 3.6 and Section 3.10 test H1 to H3b, as labelled in their headings. Section 3.4 addresses the dissociation predicted by H4, and Section 3.12 summarizes the outcome of each hypothesis.

### 3.2. Initial Discovery of Source Cues (TTFF; H1)

Participant-level TTFF on the merged-source AOI revealed a pronounced ordering of discoverability across attribution formats. The card list type (B) was discovered almost immediately upon screen onset (M = 0.96 s), followed by the inline component type (A; M = 7.34 s) and the panel type (C; M = 11.17 s), with the raw hyperlink type (D) discovered last (M = 15.65 s). A Greenhouse–Geisser-corrected repeated-measures ANOVA on raw TTFF confirmed a statistically significant condition effect, F(1.83, 40.2) = 6.40, εGG = 0.609, pGG = 0.005, partial η^2^ = 0.225, a large effect by conventional benchmarks [68]. Because raw TTFF was strongly right-skewed, the ln(1 + x)-transformed values constituted the primary test entering the Holm-adjusted family (Section 2.7, Table 7), with the raw-scale ANOVA above reported for interpretability; the effect was, if anything, stronger on the transformed scale, F(2.28, 50.1) = 11.47, εGG = 0.759, pGG < 0.001, partial η^2^ = 0.343. Holm-adjusted pairwise comparisons on the transformed values showed that top cards were discovered faster than inline chips (pHolm < 0.001, dz = 1.00), the side panel (pHolm < 0.001, |dz| = 1.09), and raw hyperlinks (pHolm < 0.001, |dz| = 1.25); no other TTFF pair survived Holm correction, corroborating the advantage of the card list type over the late-discovered formats.

Two aspects of this pattern deserve emphasis. First, the near-instantaneous discovery of the card list type is consistent with its placement above the answer body, squarely within the entry zone of the F-shaped scanning pattern [44], combined with the high visual salience of bounded card modules. Second, and contrary to the expectation embedded in H1, the panel type, although a stand-alone module occupying the largest source area of the four conditions, was discovered later than the inline component type. This A–C ordering, however, was not statistically significant after Holm correction and proved order-sensitive (Section 3.9); it is described but not interpreted substantively. Visual size alone, therefore, did not guarantee early discovery: the largest layout was not the fastest found. H1 was therefore only partially supported: visual independence accelerated discovery when it coincided with the natural entry point of reading (card list), but not when the stand-alone module was displaced to the periphery (panel). Table 6 summarizes the interpretation.

### 3.3. Depth of Processing (TDF, Fixation-Time Ratio, and Visit Structure; H2)

Depth of engagement showed a markedly different ordering from discoverability. Participant-level TDF on the merged-source AOI was highest for the panel type (C; M = 10.77 s), followed by the card list type (B; M = 9.11 s), the component type (A; M = 4.87 s), and the hyperlink type (D; M = 4.35 s). The same two-group ordering held for the share of total fixation time spent within source regions (C: 22.83%; B: 19.54%; A: 10.49%; D: 9.40%) and for cumulative fixation count (C: M = 46.39; B: M = 36.91; D: M = 16.39; A: M = 15.26); A and D did not differ significantly on any of the four primary gaze outcomes.

Layout affected each engagement outcome (Table 7). For log TDF, F(2.53, 55.6) = 12.58, εGG = 0.843, pGG < 0.001, Holm-adjusted *p* < 0.001, partial η^2^ = 0.364; top cards and the side panel each exceeded inline chips and raw hyperlinks (all pHolm ≤ 0.005, |dz| = 0.75–0.93), while B–C and A–D did not differ. The same structure held for the logit fixation-time ratio, F(2.65, 58.4) = 12.61, εGG = 0.885, pGG < 0.001, Holm-adjusted *p* < 0.001, partial η^2^ = 0.364: cards and the panel exceeded chips and links (all pHolm ≤ 0.006, |dz| = 0.74–0.96), with no B–C or A–D difference. Fixation count showed the largest omnibus effect, F(2.46, 54.1) = 19.13, εGG = 0.820, pGG < 0.001, Holm-adjusted *p* < 0.001, partial η^2^ = 0.465; again, cards and the panel exceeded chips and links (all pHolm < 0.001, |dz| = 1.06–1.14), while B–C and A–D were not significant.

At the level of gaze episodes, source-directed gaze was segmented into visits: uninterrupted runs of consecutive fixations within the merged-source AOI (Section 2.5), each visit constituting one transition from the surrounding screen into the source region. Visits were most frequent for the chips (M = 10.9 per participant vs. 10.0 for cards, 6.6 for links, and 4.3 for the panel; F(2.57, 56.5) = 14.41, εGG = 0.857, pGG < 0.001, partial η^2^ = 0.396; chips and cards each exceeded the panel, pHolm ≤ 0.001, and the links, pHolm = 0.001 and 0.029, respectively), whereas mean visit duration showed the reverse ordering (panel 3179 ms; cards 936 ms; links 652 ms; chips 441 ms; ln-scale F(2.12, 46.7) = 56.20, εGG = 0.707, pGG < 0.001, partial η^2^ = 0.719, all pairwise pHolm ≤ 0.004). Mean single-fixation durations fell within the conventional 200–400 ms band in every condition (232–319 ms). (Visit duration was quantified as the summed duration of a visit’s constituent fixations, excluding inter-fixation intervals, so each participant’s visit count multiplied by mean visit duration reproduces that participant’s TDF exactly. Condition-level products can nonetheless diverge from mean TDF (by 26% for the panel) because visit count and visit length covaried negatively across participants.) At the visit level, this characterizes the component type as a briefly glanced, frequently revisited cue rather than a reading destination, even though its individual fixations were the longest of the four conditions. The visit structure separates the two stand-alone formats rather than uniting them. Only the panel showed the rare-entry, long-episode profile, the signature of wholesale reading in which claim-level mapping is impeded. The card list was re-entered nearly as often as the chips, but with visits of intermediate length, and the chips were checked repeatedly and briefly. None of these profiles matches efficient claim-by-claim verification (Section 4.1). Consistent with a graded rather than uniform picture, only the panel, by far the densest and most displaced layout, showed elevated pupil diameter (Section 3.6); the card list did not differ detectably from the other non-panel layouts on that exploratory index. These results support H2: source UIs organized as a single semantic chunk (panel, card list) drew substantially more source-directed fixation and longer total duration of fixations than the non-chunked variants (whether this reflects engagement or mapping cost is taken up in Section 4.1). The hyperlink type showed an asymmetric profile: late discovery, with moderate episode lengths once found (M ≈ 0.7 s per visit and the second-longest single fixations at 266 ms). This pattern suggests that bare URLs fail first and foremost at attracting attention. The convergence of TDF, relative fixation time, and count indicates that B and C elicited more source-directed visual processing than A and D; it does not, by itself, establish that the processing was easier or more successful (Section 4).

### 3.4. Discoverability and Engagement Dissociated (H4)

**Figure 7:**
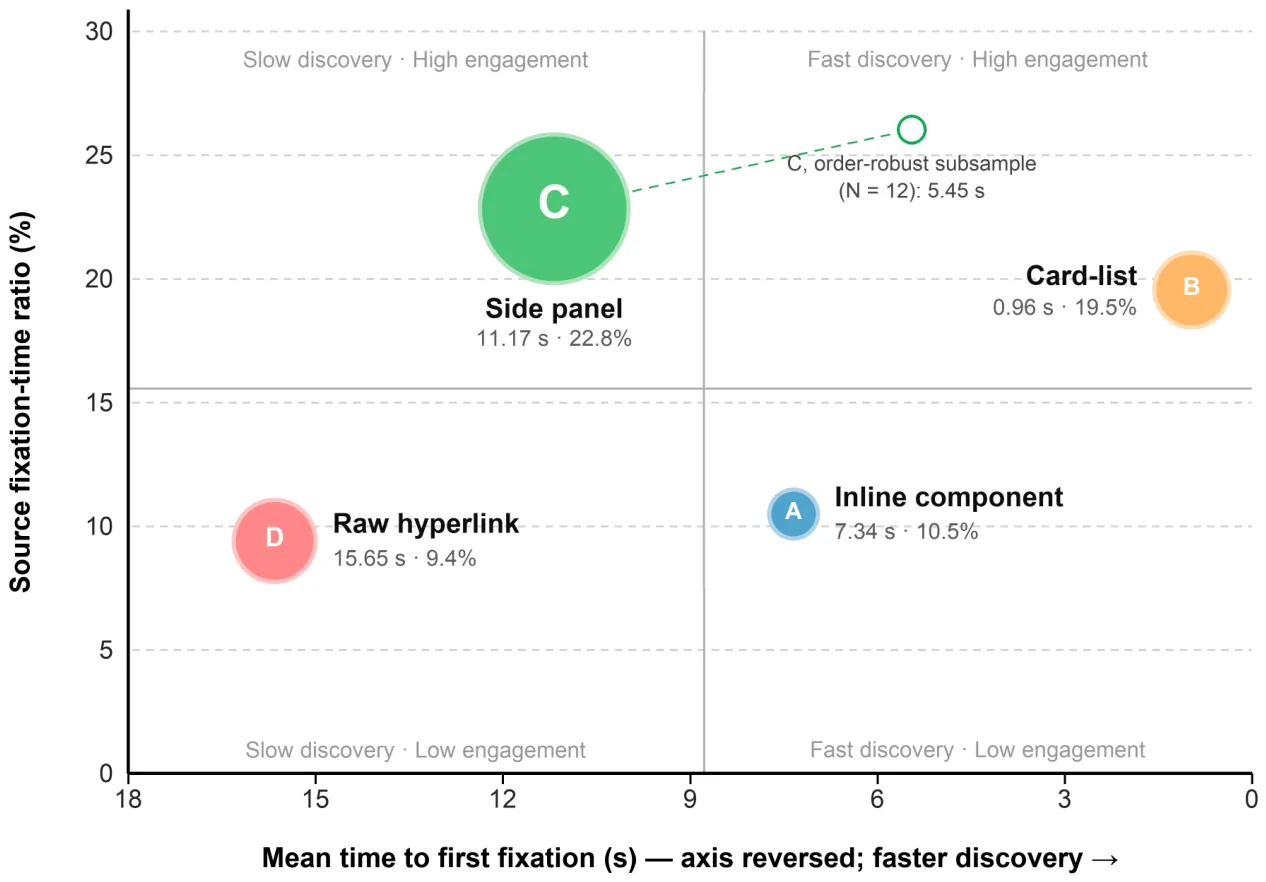
Discoverability and engagement are distinct interface outcomes. The horizontal axis is reversed, with shorter time to first fixation (faster discovery) plotting rightward, so the upper-right region corresponds to fast discovery with high engagement. Bubble area represents relative source-region area and is descriptive, not an area correction. Quadrant boundaries are grand means across the four layouts. Horizontal placement is descriptive: only the card list TTFF contrasts are significant after Holm correction, and the panel’s slow discovery is order-sensitive (leave-out M = 5.45 s, shown as the open marker; Section 3.9).

Figure 7 positions each layout by mean TTFF and mean source fixation-time ratio. The card list type occupies the high-discoverability/high-engagement region. The side panel produced the highest mean engagement but much slower discovery. The inline component was descriptively discovered earlier than links (the A–D contrast was not significant), but generated low total engagement, and raw hyperlinks were both slow to discover and low in engagement. These patterns argue against a single “visibility” construct: a layout can be prominent once noticed, yet fail to attract an early fixation or be discovered rapidly without supporting easy claim–source mapping. Participant-level correlations quantify the dissociation: within participants (pooled after centering on participant means; *n* = 92 observations from 23 participants, df = 68 after the centering; descriptive, not confirmatory), discovery speed and total dwell were only moderately (and inversely) coupled, r(TTFF, TDF) = −0.40, 95% CI [−0.58, −0.19], *p* < 0.001, and neither gaze metric tracked trust ratings, r(TTFF, trust) = −0.20, 95% CI [−0.41, 0.04], *p* = 0.102, and r(TDF, trust) = 0.10, 95% CI [−0.13, 0.33], *p* = 0.388. Attention moved without moving judgment. (Part of the negative TTFF–TDF coupling is mechanical: under a fixed 60 s exposure, later discovery leaves less time in which dwell can accumulate.)

### 3.5. Area-Normalized Attention Efficiency

**Figure 8:**
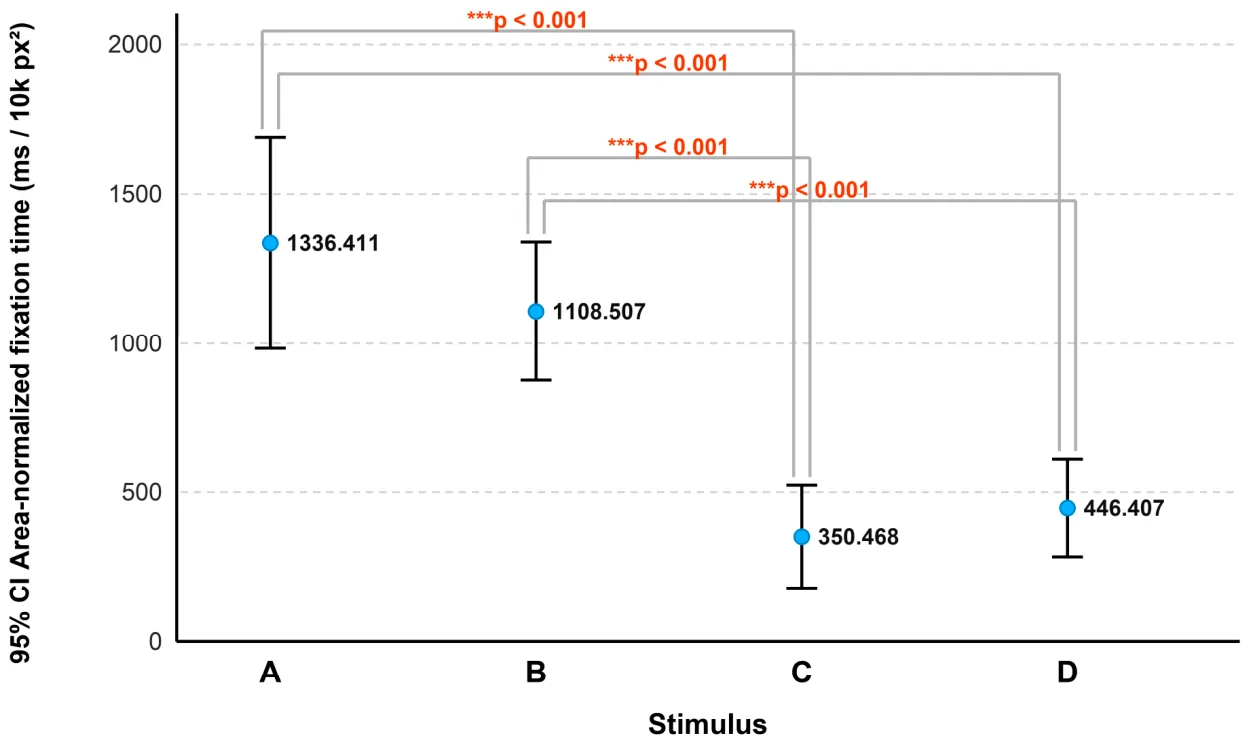
Area-normalized source fixation time (source fixation duration divided by merged source-region area, scaled to ms per 10,000 px^2^) by source-attribution layout (A: inline component; B: card list; C: side panel; and D: raw hyperlink). Dots are condition means, and whiskers are within-participant 95% confidence intervals (Cousineau–Morey correction); brackets denote significant Holm-adjusted pairwise comparisons (*** *p* < 0.001).

Because the source AOIs differed greatly in area, with the panel type occupying roughly an order of magnitude more screen real estate than the inline component (Table 2), raw TDF conflates attention with size. An area-normalized source fixation time (fixation duration divided by AOI area, scaled to ms per 10,000 px^2^) reversed the raw ordering (Figure 8): the component type was most efficient (1336), closely followed by the card list type (1109), with the hyperlink (446) and panel (350) types markedly lower. A Greenhouse–Geisser-corrected repeated-measures ANOVA revealed a significant and large condition effect, F(1.74, 38.2) = 17.34, εGG = 0.579, pGG < 0.001, partial η^2^ = 0.441. Because the merged source-region area is constant within each condition, this analysis is a deterministic rescaling of the dwell data rather than independent evidence; it is reported to make the size-adjusted ordering and its magnitude explicit (Section 4.4). Holm-adjusted pairwise comparisons showed that the component and card list types each attracted significantly more fixation time per unit area than the panel and hyperlink types, whereas the component–card and panel–hyperlink contrasts were not significant.

This analysis sharpens the interpretation of Section 3.3. The descriptive inversion does not invalidate the panel’s high raw TDF; rather, it indicates that the panel’s attention was distributed across a much larger source region, whereas the component and card list types concentrated gaze in far less screen space. The panel type is thus best characterized as a much-seen UI whose raw dwell advantage is partly a consequence of its area, whereas the component and card list types are efficient UIs that concentrate visual attention despite small footprints. We treat the area-normalized index as a complementary description rather than a definitive area correction because source area was intentionally designed and perfectly confounded with layout, so density cannot isolate a “pure” layout effect and is not used to rank overall effectiveness (Section 4.4). Nevertheless, the pattern shows that making sources visible and making them spatially efficient are distinct design achievements: among the four typologies, only the card list type accomplished both.

### 3.6. Cognitive Load (Exploratory Pupillometry; H3a)

**Figure 9:**
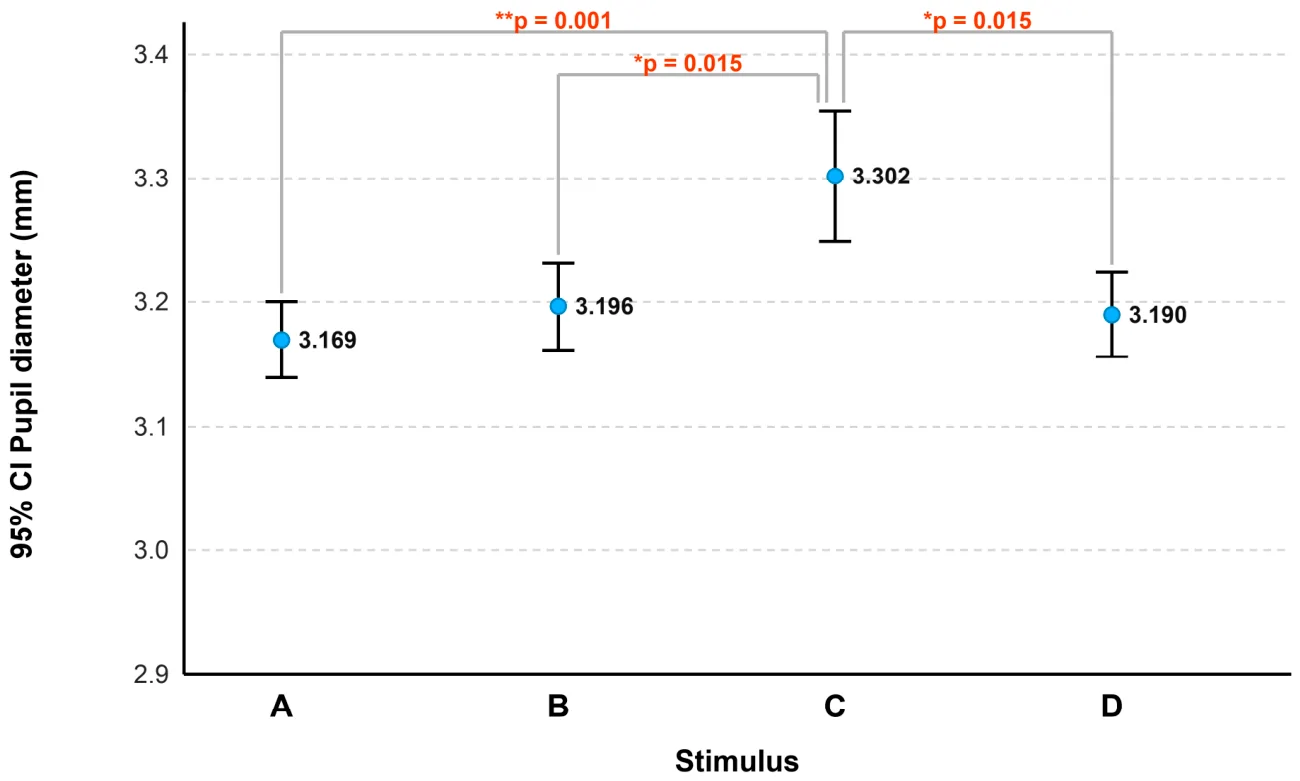
Mean whole-fixation pupil diameter (mm) by source-attribution layout (A: inline component; B: card list; C: side panel; and D: raw hyperlink). The y-axis is truncated (2.9–3.4 mm) and does not start at 0; the significant between-condition differences (0.10–0.13 mm) should be read against the between-participant SD of 0.32–0.35 mm. Dots are condition means, and whiskers are within-participant 95% confidence intervals (Cousineau–Morey correction); brackets denote significant Holm-adjusted pairwise comparisons (* *p* < 0.05, ** *p* < 0.01).

Average whole-fixation pupil diameter, taken as an exploratory physiological index of processing load [45,46], separated the side panel from the other three layouts (Figure 9). Diameter was quantified as the mean of Tobii’s filtered binocular pupil signal across all fixation samples within each 60 s stimulus interval, matching the whole-fixation definition in Table 3: condition means were 3.17 mm (SD = 0.32) for the inline component, 3.19 mm (0.33) for the hyperlink, 3.20 mm (0.35) for the card list, and 3.30 mm (0.35) for the panel. A repeated-measures ANOVA confirmed the condition effect, F(2.17, 47.8) = 9.92, εGG = 0.724, pGG < 0.001, partial η^2^ = 0.311, corroborated nonparametrically (Friedman χ^2^(3) = 18.29, *p* < 0.001); Holm-adjusted pairwise comparisons showed that the panel exceeded every other layout (vs. A: pHolm = 0.001, dz = 0.93; vs. B and D: pHolm = 0.015, dz ≈ 0.70), with no other pair differing. Four artifact accounts (near-constant stimulus luminance, gaze-angle-dependent pupil foreshortening, the unavailability of baseline correction, and the panel-specific column reflow) are examined in detail in Appendix C. None fully explains the effect: the panel’s elevation persisted when only fixations outside the source regions were analysed, although residual contributions cannot be excluded and the load interpretation remains exploratory. Contrary to H3a, the familiar hyperlink format was not processed with reliably less effort, as the three non-panel layouts were statistically indistinguishable. Only the information-dense side panel elevated processing load, converging with the mapping-cost account of its long dwell (Section 4.1).

### 3.7. Moderating Role of User Characteristics (Exploratory)

These stratified observations are hypothesis-generating only: with *N* = 23 divided into small, imbalanced subgroups, no inferential tests were conducted, and the tendencies summarized below and detailed in Appendix D are descriptive patterns awaiting replication in adequately powered samples (Section 2.7).

In brief, low-frequency users tended toward broader search and longer dwell on most source regions, whereas high-frequency users showed efficient existence-checking scans except on the information-dense panel, which they exploited longer; multi-platform users discovered source cues faster and dwelled longer on structured source modules; and design majors gravitated toward standard-conforming, visually polished cues, whereas non-design majors treated chunked source structures as pragmatic verification tools. The full stratified descriptions are reported in Appendix D.

### 3.8. Heatmap Analysis

**Figure 10:**
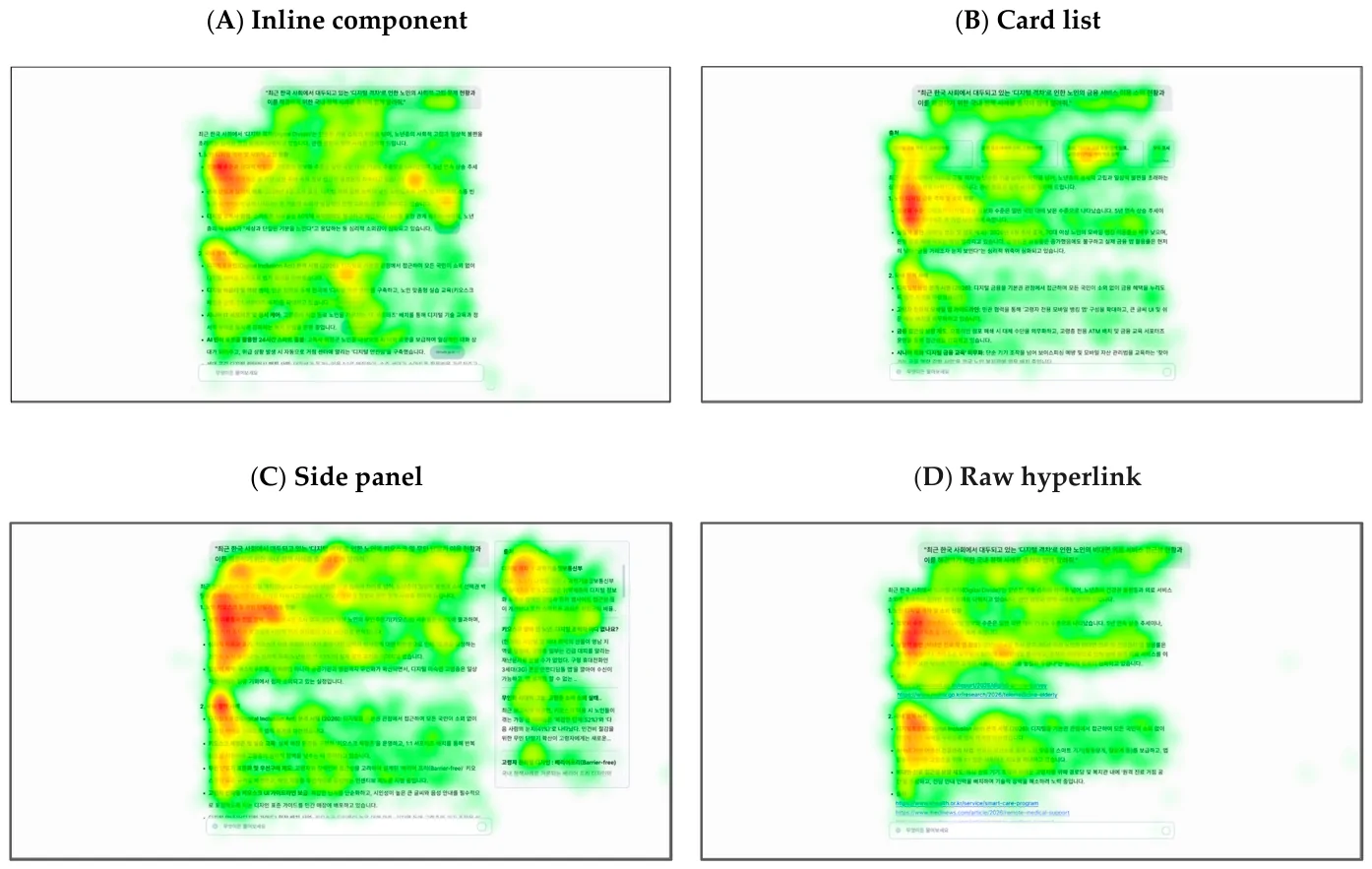
Accumulated gaze heatmaps over the full 60 s exposure for Stimuli (**A**–**D**) (cumulative fixation duration). (**A**): inline component; (**B**): card list; (**C**): side panel; and (**D**): raw hyperlink. Warmer regions denote longer accumulated dwell. The Korean-language stimuli are reproduced as presented to participants.

Accumulated heatmaps over the full 60 s exposure exhibited the canonical F-shaped baseline [44] modulated by each attribution format (Figure 10).

In Stimulus A, dense fixation clusters on the left and centre of the body text were accompanied by clear fixation anchors on the chip components at line ends, indicating that participants registered source cues in the course of reading. In Stimulus B, the most intense concentration appeared on the card grid, consistent with the near-immediate capture indexed by TTFF (M = 0.96 s; Section 3.2); because cumulative heatmaps do not encode temporal order, sequencing claims rest on the fixation-timing data rather than on the map itself. In Stimulus C, a dense high-intensity region formed over the right-hand panel, the spatial footprint of the long, infrequent reading episodes documented in Section 3.3; as argued in Section 4.1, this concentration is better read as effortful or impeded claim–source mapping than as voluntary deep comparison. In Stimulus D, fixations fragmented into small clusters over the blue underlined links listed beneath each answer section, indicating sequential acquisition of sources during top-down reading but also gaze fragmentation caused by the links’ small visual footprint.

### 3.9. Sensitivity to Distribution and Presentation Order

Nonparametric Friedman tests on the raw outcomes confirmed condition effects for TTFF, χ^2^(3) = 23.88, *p* < 0.001; TDF, χ^2^(3) = 21.21, *p* < 0.001; fixation-time ratio, χ^2^(3) = 21.63, *p* < 0.001; and count, χ^2^(3) = 32.00, *p* < 0.001. Participant-fixed sensitivity models that included presentation position also retained strong condition effects for every transformed outcome (all *p* < 0.001). However, presentation position itself was significant in every model (all *p* < 0.001). The layout pattern is therefore robust to adjustment in this sample, but the order effect is substantive and reinforces the need for prospective counterbalancing in replication.

As a further check aimed at the largest allocation imbalance (condition C appearing first for 11 of 23 participants), we repeated the Friedman tests on the participant-level outcomes (Section 2.6) after excluding those 11 participants (*N* = 12). Every condition effect persisted (TTFF: χ^2^(3) = 14.42, *p* = 0.002; TDF: χ^2^(3) = 15.90, *p* = 0.001; fixation-time ratio: χ^2^(3) = 15.90, *p* = 0.001; fixation count: χ^2^(3) = 17.31, *p* < 0.001; the identical values for TDF and the fixation-time ratio reflect identical within-participant rank orderings in this subsample): the card list was still discovered first (M = 1.03 s), with the hyperlink still last (M = 22.83 s), and the panel still attracted the deepest engagement (fixation-time ratio M = 26.03%; total dwell M = 12.40 s). The estimate most sensitive to allocation was the panel’s discovery latency, which shortened from 11.17 s to 5.45 s once its overrepresentation in the first and slowest position was removed. The panel-versus-component TTFF ordering should therefore not be over-interpreted, whereas the primary discovery contrast (cards first, links last) and the engagement ordering were order-robust. Descriptively, the position effect resembled practice gains: pooled across layouts, mean TTFF fell from 18.46 s at the first position to 3.65–5.03 s at the last two positions, while the source fixation-time ratio rose from 13.24% to 18.08%.

### 3.10. Subjective Evaluations (H3b)

Descriptive statistics for the four survey constructs are reported in Table 8 (*N* = 23 per condition; 92 responses; no missing data). All means clustered in a mildly positive band (4.35–5.39 on the seven-point scale). No single layout dominated: readability was descriptively highest for top cards (M = 5.39), information-seeking flow for inline chips (M = 5.22), trust impression for the side panel (M = 5.09) and inline chips (M = 5.00), and intention to use for chips and the panel (both M = 5.17). Their ordering differs from the gaze ranking: the two layouts receiving the most source-directed attention were not consistently preferred or trusted most.

Repeated-measures ANOVAs (Greenhouse–Geisser-corrected), treating layout as the within-participant factor, revealed no statistically significant differences among the four UI types on any construct: readability, F(2.34, 51.4) = 1.13, εGG = 0.778, pGG = 0.338, partial η^2^ = 0.049; information-seeking flow, F(2.23, 49.0) = 1.91, εGG = 0.743, pGG = 0.155, partial η^2^ = 0.080; perceived trustworthiness, F(2.67, 58.8) = 0.79, εGG = 0.891, pGG = 0.493, partial η^2^ = 0.035; and behavioural intention, F(2.44, 53.7) = 0.59, εGG = 0.813, pGG = 0.590, partial η^2^ = 0.026. Friedman tests agreed (all χ^2^(3) ≤ 5.64, *p* ≥ 0.130), Holm-adjusted paired contrasts yielded no significant pairwise differences (all pHolm ≥ 0.327), and between-participant variance, rather than UI format, accounted for the bulk of score variance. Because each construct was measured with a single item, these comparisons should be read as descriptive of this sample rather than as evidence of equivalence (Section 5). In particular, the hyperlink format showed no trustworthiness advantage over the other layouts, providing no support for H3b. Formal equivalence tests reinforce this caution: two one-sided tests on the trustworthiness item (TOST; [74,75]) established equivalence within a lenient ±1-point bound for only two of the six layout pairs (component–panel pTOST = 0.035, card–hyperlink pTOST = 0.016) and for no pair within ±0.5 points; the panel–hyperlink difference in particular could not be bounded below one scale point (Mdiff = 0.52, 90% CI [−0.13, +1.18]). The data are thus compatible with no difference but do not demonstrate equivalence.

Three descriptive patterns nonetheless merit attention. First, the component type recorded the lowest readability (M = 4.78) but the highest information-seeking flow (M = 5.22), whereas the panel type showed the reverse profile (readability M = 5.22; flow M = 4.35), revealing a trade-off between the visual comfort of a layout and its efficiency in guiding search: dense inline annotation compromises legibility while accelerating claim-by-claim navigation, and a spacious aggregated panel does the opposite. Second, perceived trustworthiness and behavioural intention indicator tracked each other almost perfectly across conditions, suggesting that participants did not psychologically separate trust attitude from reuse intention but judged them as a single dimension. Third, the profile of means clustered structurally similar architectures together: the sentence- or claim-anchored A and the content-preview-rich C patterned above the aggregated-without-mapping B and the bare-link D on the two trust constructs. This pattern descriptively suggests that users perceived UIs through underlying design architecture rather than surface details, although no contrast reached significance. Response dispersion also differed informatively: the component type showed the largest between-participant disagreement on readability (SD = 1.68), whereas the card list type showed the strongest consensus (SD = 0.99), marking inline annotation as a polarizing design.

### 3.11. Post-Interview Preferences and Qualitative Themes

**Figure 11:**
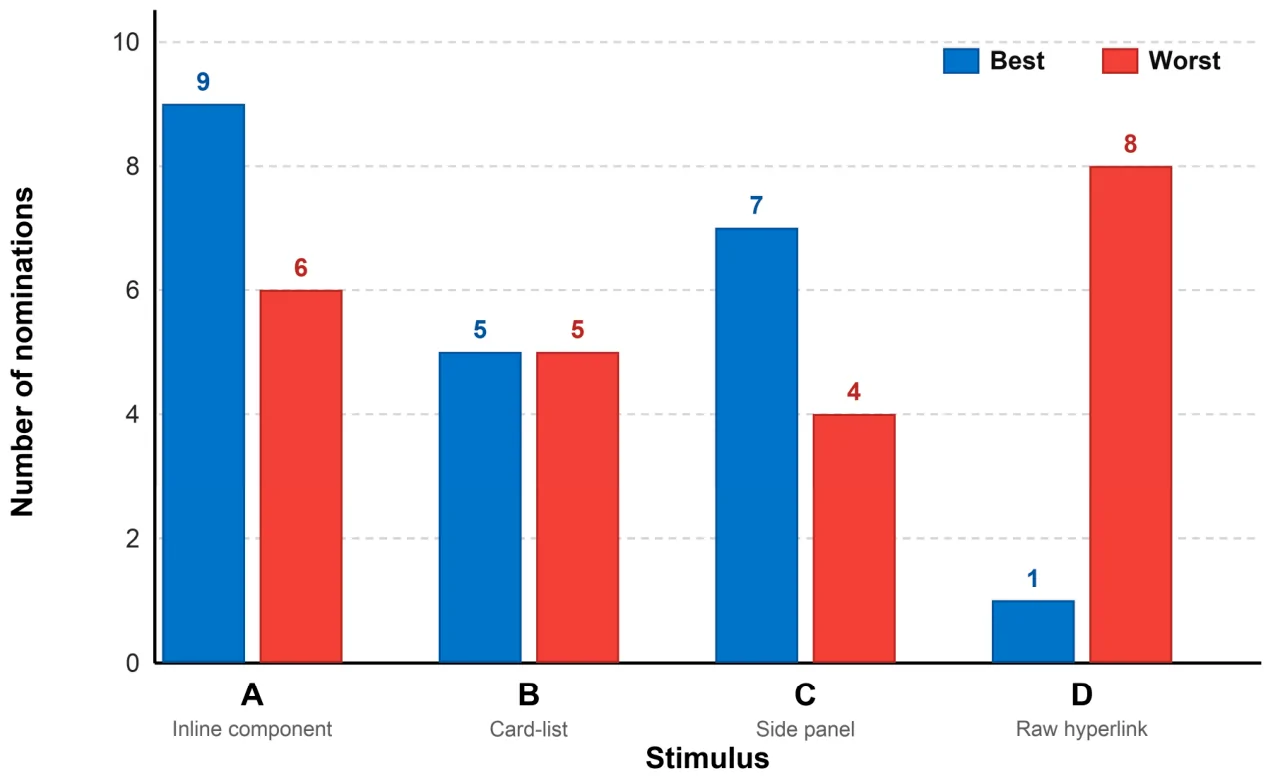
Best-UI (blue bars) and worst-UI (red bars) nomination counts by source-attribution type from the post-interview (22 of 23 participants; one participant declined to nominate a best or worst format and is excluded from the count, and one nominated two formats as worst). The most-preferred format, the inline component type, attracted roughly half the raw source dwell of the stand-alone formats.

All 23 participants answered affirmatively that the manner of source attribution influenced their trust, confirming the manipulation’s subjective relevance. When asked to nominate the best UI, the modal preference was the component type (A; 9 nominations), followed by the panel (C; 7), the card list (B; 5), and the hyperlink (D; 1). The most preferred format thus attracted roughly half the raw dwell of the stand-alone formats. The worst-UI nominations partially inverted this ranking: the hyperlink type drew the most (D; 8), followed by the component (A; 6), card list (B; 5), and panel (C; 4). Formal tests qualify these counts: neither nomination distribution departed significantly from uniformity (best: χ^2^(3) = 6.36, *p* = 0.095; worst: χ^2^(3) = 1.52, *p* = 0.68), whereas the focal contrast was reliable, with the component type nominated best more often than the hyperlink type (9 vs. 1; two-sided binomial *p* = 0.021). Combining the two questions into a net preference score (best minus worst nominations) makes the balance explicit: the component and the panel tie at +3 (9 − 6 and 7 − 4), the card list sits at 0 (5 − 5), and the hyperlink type at −7 (1 − 8). The extremes were reversed, in that the seldom-preferred hyperlink drew the most worst votes and the long-dwell panel the fewest. The most-preferred component type was simultaneously the second-most-frequent worst choice, marking it as the most polarizing layout. This polarization is consistent with its condition-highest dispersion on readability and information-seeking flow, and its marginally highest dispersion on the two trust-related constructs as well (trustworthiness SD = 1.48, behavioural intention SD = 1.53, in each case the largest of the four layouts; Table 8; Figure 11). (One participant declined to nominate a single best/worst and was excluded from the count; one nominated two formats, A and D, as worst.)

Thematic coding of interview comments and sketches converged on four cross-cutting demands, whose prevalence by UI is summarized in Table 9. For the component type, participants valued the sentence-level anchoring (“each line has its own source, so it feels trustworthy,” P6, P19) but criticized the absence of pre-click information (“until you click, you cannot know what it is,” P18) and the chips’ bulky footprint disrupting line spacing (P2, P23). For the card list type, the dominant complaint was the absence of body-to-card mapping (“which line corresponds to which card?,” P18) and the placement of sources above the content, which several participants experienced as stealing attention before reading (“sources should not come before the topic,” P5). For the panel type, the content preview was praised as directly supporting comprehension and trust (“showing the content directly raises the credibility of the information,” P15), yet the lack of claim-level matching undermined that benefit (“the sources sit off to the side and never match the body—before I can even trust it, I cannot even map the information,” P8), and the dense text was experienced as visually suffocating (P1, P13). For the hyperlink type, 12 of 23 participants independently demanded titles or institutional metadata in place of raw URLs (“a bare blue address tells me nothing before I click,” P16, P20), and several reported prior experiences of broken or irrelevant links that preloaded distrust of the format (P3, P15).

Table 10 integrates the quantitative gaze signatures with the qualitative strengths and barriers reported for each layout.

### 3.12. Summary of Hypothesis Tests

Table 11 summarizes the verdict on each hypothesis together with its key evidence.

Table 12 consolidates the condition-level outcomes: the mean values of the four primary gaze metrics, the exploratory pupil index, and the post-interview preference counts for each layout.

## 4. Discussion

### 4.1. The Attention–Preference Gap

How, then, does source-attribution visualization shape user attention and preference? For attention, the four layouts produced two distinct orderings. The card list was discovered almost immediately and the raw hyperlinks last, whereas the side panel and the card list sustained the deepest engagement once found. Preference tracked neither ordering: the inline chips attracted roughly half the dwell time of the stand-alone formats, yet were nominated as the best design most often.

The central finding of this study is a systematic dissociation between where users look and what users prefer. The stand-alone formats commanded the longest and most repeated fixation, yet the format users most often nominated as best was the inline component type, whose raw dwell was roughly half that of the stand-alone formats. This preference axis rests primarily on the forced-choice nominations, in which the component type was nominated best significantly more often than the hyperlink type (nine votes versus one; binomial *p* = 0.021), although the four-way nomination distribution itself did not depart significantly from uniformity (Section 3.11), and on the absence of gaze–trust coupling (Section 3.4). The net preference score reported in Section 3.11 fixes the scope of this dissociation. Because best and worst nominations offset for the panel, the component and the panel tie on net preference (+3 each). The divergence is therefore carried chiefly by the component type, which attracted near-minimal dwell yet the most best nominations, whereas attention and preference converge for the panel, which ranks high on both, and for the raw hyperlinks, the least-dwelled and the least-preferred format. The flat single-item Likert profiles corroborate rather than carry the dissociation (Section 3.10 and Section 5). Triangulating gaze data with interview evidence offers a resolution. The prolonged dwell on the card and panel regions is more consistent with the cost of mapping, that is, working out which claim each source supported, than with positive engagement with the sources themselves. The panel showed rare but prolonged reading episodes and the card list frequent re-entries of intermediate length, neither matching efficient claim-by-claim lookups (Section 3.3). The side panel illustrates the ambiguity most clearly: it offered the richest source summaries, so some of its gaze plausibly reflects useful inspection, yet the most frequent panel request was bidirectional highlighting between the answer and source entries, indicating that repeated transitions and prolonged fixation also reflect the effort required to infer correspondence. Long fixation duration, often read as an index of interest in consumer eye-tracking research, here indexed friction as much as engagement. This is a general interpretive risk: fixation duration indexes visual processing demand, not valence or success [31,32,48]. Treating more gaze as automatically better would misread both the panel and the chips: the inline-chip design produced low-source TDF but was praised for claim-level traceability. Eye tracking is most useful when it identifies where processing occurs and qualitative or task-performance measures explain why. The mapping-cost account should accordingly be read as the interpretation most consistent with the present data rather than as a measured construct. Separating it from genuine engagement (e.g., with claim–source lookup tasks, comprehension probes, or cued retrospective think-aloud) is a direct test for future work. More broadly, because the stimuli were static and non-clickable, this study measured the visual preconditions of verification (noticing and attending to sources) rather than verification acts themselves.

The dissociation extends the citation-trust literature in a process direction. Prior work established that the presence of citations inflates trust regardless of their validity [6,22], that generated citations frequently fail to support their claims [7], and that users verify almost nothing [1]. Our gaze data show why format matters within that regime: formats differ enormously in whether they are even noticed (a 16-fold TTFF spread) and in how they convert screen area into attention, yet none of these attentional differences propagated into significant differences in self-reported trust. In terms of prominence–interpretation theory [10], the manipulations powerfully altered prominence. Interpretation, the credibility meaning users assigned, remained largely governed by a different variable: whether the UI let them connect each claim to its evidence.

### 4.2. Discoverability and Verification Efficiency Are Distinct Design Goals

Making sources visible and making them efficiently verifiable emerged as separable achievements. The card list type uniquely combined near-instant discovery, sustained dwell time, and high area-normalized efficiency, giving it the most balanced attention profile of the four (Table 12; Section 3.5). It was the only layout that was separated from every other condition in TTFF. Its placement above the answer and its grouping into a visually bounded block made the existence of sources immediately legible. Yet the interviews reveal why discoverability is insufficient: participants often could not tell which card supported which claim. The card block attracted attention before or apart from the relevant sentence, creating a source overview without an evidentiary mapping. A design can therefore optimize TTFF and still leave the central verification question unresolved: “what, exactly, does this source support?” A quickly noticed but poorly mapped citation may function more as a credibility badge than as a verification tool [6].

The panel type maximized raw visibility but at the lowest spatial concentration of the four layouts and, on the exploratory pupillometric index, at elevated cognitive load, functioning as a much-seen rather than a well-used UI (Table 12; Section 3.5 and Section 3.6). The component type converted the smallest footprint into the highest attention density (Section 3.5) and the descriptively best information-seeking flow ratings, but its readability cost and domain-only pre-click chips divided users. The hyperlink type failed primarily at the attention-capture stage: it was found last and attracted the least dwell (both descriptively; its contrasts with the inline component were not significant), it was sampled only in brief visits once found (Section 3.3), and its absent metadata blocked interpretation. Designers should therefore treat discoverability (placement within the F-pattern entry zone; bounded modular salience) and verification efficiency (compact, claim-anchored, information-scented cues) as independent requirements to be jointly satisfied. In practice, the two requirements call for different mechanisms within the same interface: an overview cue placed in the natural entry zone, so that the existence of sources is legible at a glance, and compact claim-anchored markers that keep provenance attached to the sentence being read. Each commercial archetype studied here satisfied at most one of the two, which is precisely the gap the two-stage architecture of Section 4.6 is designed to close. These findings complement recent evidence that source presentation changes interaction and persuasion in conversational search [27], while extending it with direct pre-click measures.

### 4.3. Pre-Click Source Cues and Provenance Mapping as Trust Infrastructure

Across all four formats, the two qualitative demands voiced by the largest numbers of participants were (i) source identifiability before clicking, that is, titles and institutional provenance in place of chips or raw URLs (12 of 23 for the hyperlink type alone) and (ii) explicit claim–source mapping (10 of 23 for the panel type; 8 of 23 for the card type). Both demands are, at bottom, requests for verifiability without leaving the answer [76]: participants wanted to assess a source’s authority (“is this from the Financial Supervisory Service or a blog?”) and its relevance (“does this link actually contain the quoted claim?”) at a glance. When mapping was absent, several participants reported that unmatched sources actively increased suspicion of fabrication (“if I cannot see where it came from, I would suspect the sentence was made up”). This is a striking inversion: the mere presence of citations, shown by prior work to inflate trust [6], can undermine trust when their structural connection to claims is severed. Given that LLMs are prone to unfaithful generation [5], that attribution UIs are among the few levers available to promote responsible trust calibration [17,21], and that displaying sources has recently been shown experimentally to reduce reliance on incorrect LLM answers [77], sentence-level provenance mapping should be understood not as a stylistic option but as trust infrastructure.

### 4.4. AOI Geometry Is Part of the Treatment, Not Merely Noise

The four source regions differed greatly in area, and it is tempting to divide dwell time by pixels and treat the result as a fairer comparison. That move is only partially defensible. The large panel was intentionally designed to signal importance and provide more content; removing its area “advantage” also removes part of the interface intervention. Element size is among the strongest predictors of accumulated gaze on web page elements [37], a conclusion consolidated by meta-analysis, in which surface size, salience, and position shape gaze allocation as strongly as, or more strongly than, task factors [78], and size effects on attention are element-dependent rather than uniform: attention to text grows roughly in proportion to its surface, whereas pictorial elements capture attention largely independent of their size [56]. Moreover, the assumption that expected fixation time grows linearly with AOI area is not established: a very large panel may contain substantial unused white space, while a small badge may compress all informational content into a few pixels. Empirically, the size–attention relationship is logarithmic rather than linear, with diminishing returns for larger elements [73,79], and the attention an element receives further depends on the size and proximity of competing display elements [80]. Accordingly, our primary results retain raw TDF and fixation-time ratio, which capture the total attention generated by the complete design, and the area-density values are secondary, answering a different question: how concentrated was fixation time within the allocated source space? This dual-reporting stance follows methodological guidance to normalize AOI measures by area when regions differ in size [53] and a long lineage of per-unit-area attention measures: small advertisements maximize fixations per unit area even though larger ones attract more total attention [65,81], and duration-weighted fixation maps and coverage indices likewise express gaze as a density over stimulus area [66,82,83]. Most recently, an eye-tracking study of generative AI search pages reported raw fixation time alongside fixation time normalized by region word count, with the two lenses yielding complementary conclusions [41]. This distinction (total attention vs. spatial concentration) is more informative than treating normalization as a correction. A future factorial study should cross layout structure with controlled size to separate these effects experimentally.

### 4.5. Expertise Shapes Verification Strategy

The stratified analyses suggest, descriptively and given the small and imbalanced subgroups (Section 2.7 and Section 3.7), that source-verification behaviour may not be homogeneous across users. Less-frequent AI users appeared to search more broadly and read source regions more exhaustively, consistent with an unautomatised credibility-checking routine, whereas daily users deployed schema-driven, existence-checking scans, except when a high-density panel afforded genuine context comparison, which they exploited more than novices did. Multi-platform users behaved like perceptual experts, locating source cues faster and interrogating structured source modules longer. Design majors gravitated toward standard-conforming, visually polished cues, while non-design users treated chunked source structures as pragmatic verification tools. For practitioners, this heterogeneity implies that a single attribution format is unlikely to serve all user segments equally and that adaptive or layered disclosure (compact anchors by default, expandable evidence on demand) may reconcile the strategies observed.

### 4.6. A Verification-Oriented Design Pathway and Design Principles

**Figure 12:**
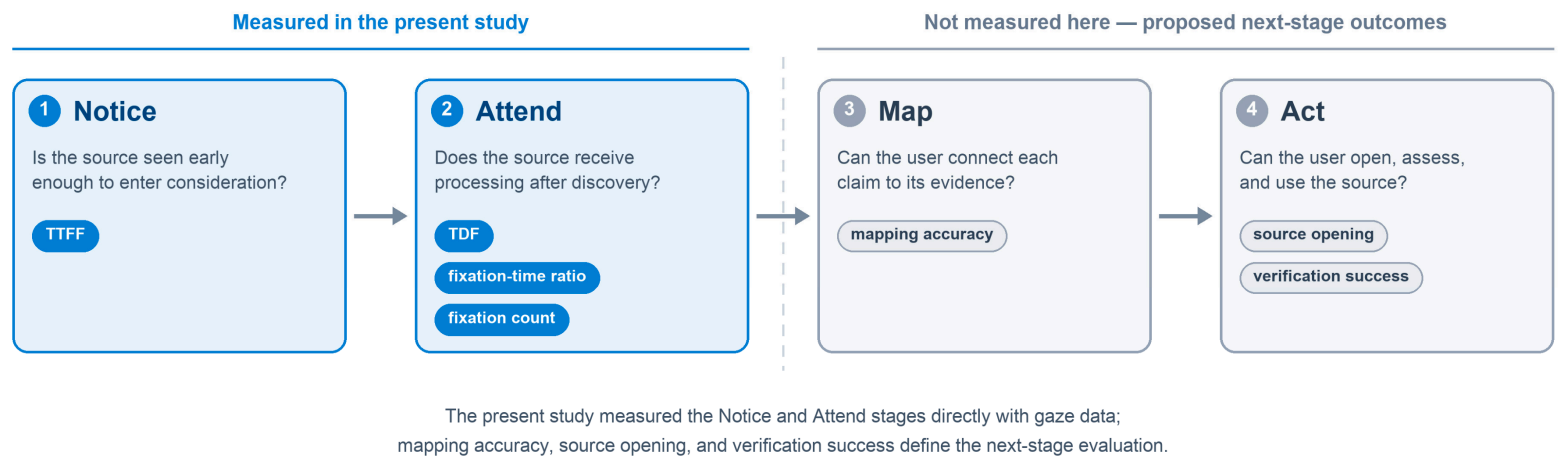
Verification-oriented pathway for evaluating source interfaces. Mapping accuracy, source opening, and verification success were not measured in the present study and are proposed as essential next-stage outcomes.

Synthesizing the quantitative and interview findings, we propose evaluating source interfaces as a sequence of four stages: Notice, Attend, Map, and Act (Figure 12). Notice asks whether the source becomes visible early enough to enter consideration and can be evaluated with TTFF. Attend asks whether the source receives processing after discovery and can be characterized with TDF, relative fixation time, and count. Map asks whether the user can correctly connect a claim to the evidence that supports it. Act asks whether the user can open, assess, and use the source to verify the claim. The present study measured the first two stages directly; mapping accuracy and verification success are essential next-stage outcomes.

**Figure 13:**
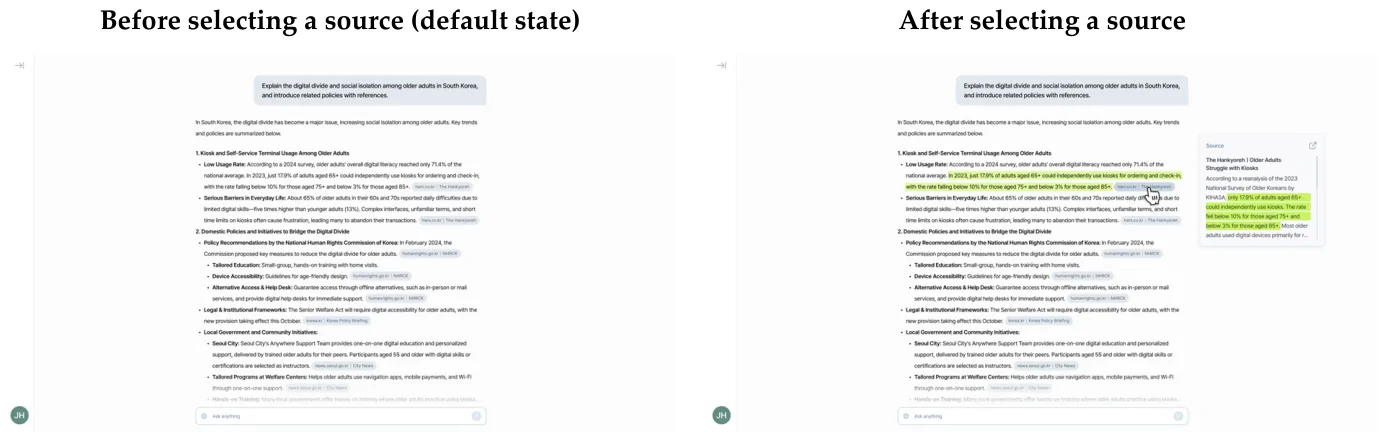
Proposed verification-oriented interface concept before (**left**) and after (**right**) a source chip is selected. The prototype augments the preferred inline component format with pre-click source identification (institution and title), explicit bidirectional claim–source highlighting, and an in situ original-content preview, addressing the interview-derived barriers summarized in Table 9. The refined chip is height-matched to the body-text line, addressing the complaint that the original chips disrupted line spacing with their bulky footprint (Section 3.11).

Three concrete design principles follow from the converging quantitative and qualitative evidence and were embodied in a refined (To-Be) prototype built on the component-type skeleton that users preferred (Figure 13). The prototype itself has not yet been empirically evaluated; it is offered as a design proposal that operationalizes the three principles and requires future experimental validation. First, sufficient pre-click identification: each inline anchor exposes the source’s institution and title in a slimmed chip whose height, weight, and background contrast were adjusted to resolve the readability complaints directed at the original component design. Second, explicit claim–source mapping: selecting a source highlights, in the body text, the exact passages it supports (and vice versa), eliminating the mapping cost that inflated dwell on the aggregated formats. Third, in situ content verification: an attached preview surface exposes the relevant excerpt of the original document, with a link out to the full source, so that users can confirm that the cited material actually contains the claimed content without leaving the chatbot.

Concretely, these principles combine into a single continuous interaction in the refined prototype (Figure 13). In its default state (Figure 13, left), every claim terminates in a slim inline chip that already names the cited outlet and its domain, so that a reader can identify the provenance and judge whether a claim warrants closer scrutiny before any click. Selecting a chip (Figure 13, right) opens a source panel beside the answer that names the outlet and the document title, renders an in situ excerpt of the original article, and highlights within that excerpt the exact sentence that grounds the adjacent claim (in the illustrated case, the passage reporting that kiosk-based ordering and payment were possible for only 17.9% of adults aged 65 and older), while the supported claim is highlighted reciprocally in the answer body, so that the claim–source correspondence is made explicit in both directions. A single link control in the panel then opens the full source for readers who want the complete context. Verification, therefore, unfolds in three low-friction actions: read the chip in place, open the panel to check the highlighted evidence, and, only when needed, follow the link to the original. In this way, its cost scales with a reader’s demonstrated need rather than being paid up front for every citation.

Generalized beyond a single prototype, these principles yield a two-stage attribution architecture. The first stage provides a persistent, semantically labelled, claim-adjacent cue: a compact source name, organization, or document type linked by an index or colour to an overview, combining the top cards’ discoverability with the inline chips’ traceability. The second stage progressively reveals richer metadata (summary, author, date, passage, and direct external access) while highlighting the supported claim and the selected source in both directions, combining the panel’s information richness with lower default density. Raw URLs should be replaced by meaningful link labels. The objective is not to maximize gaze or trust: it is to make uncertainty and provenance inspectable at proportionate cost, so users can increase or decrease reliance in response to evidence. This orientation aligns with work on appropriate reliance and cognitive forcing [17] and shifts source design from decorative transparency toward verifiable interaction. Collectively, these principles aim to convert citations from decorative trust cues into low-friction verification affordances, relieving users of the burden of manually cross-checking every claim while preserving, rather than short-circuiting, their agency as verifiers.

## 5. Limitations and Future Research

### 5.1. Sample, Measurement, and Statistical Power

Several limitations qualify the present findings. First, the sample (*N* = 23; 18 women, 5 men, all in their twenties) consisted of university students in design-adjacent and engineering programmes, a young, digitally fluent population of habitual AI users with a predominantly female composition. Generalization to older, less AI-experienced, or more gender-balanced populations, therefore, remains untested. While the sample size is well justified for gaze-pattern identification by the heatmap stability curve [59] and is comparable to established eye-tracking studies [60,61,62], the single-item Likert measures and modest N limit the power of the subjective comparisons, and the null survey effects (partial η^2^ = 0.03–0.08) should be interpreted as an absence of detected differences rather than evidence of equivalence. In addition, the post-stimulus items were administered verbally in the experimenter’s presence, so demand characteristics and socially desirable responding cannot be excluded, a risk that bears most directly on the trustworthiness item. Future work should pair validated multi-item scales with the same gaze protocol. The sensitivity calculation in Section 2.1 makes this concrete: the minimum detectable effect is f ≈ 0.27–0.31, and the observed gaze effects (f ≈ 0.54–0.93) lie far above this bound, whereas the survey effects (f ≈ 0.16–0.29) lie at or below it. Replication with larger, demographically broader samples (including older adults and low-AI-literacy users, for whom source verification stakes are arguably highest) is warranted. Extending attribution interfaces to older users will also require design accommodation, not merely resampling. Participatory design work with older adults documents wide variation in physical, cognitive, and interaction abilities, which argues for adaptable and multimodal presentation of source cues [84].

### 5.2. Design Confounds: Topic, Order, and Source Count

Second, layout was not fully crossed with content: each attribution format was paired with a fixed answer subtopic, so format and topic are partially confounded. This reflected a deliberate trade-off. Presenting an identical answer in all four conditions would have introduced severe carryover: after the first exposure, later trials would index rereading of known content rather than first-encounter search, deflating TTFF and dwelling in a layout-independent way. Content was therefore varied to protect the ocular measures from practice and familiarity effects. To bound the resulting variation, the four screens were not unrelated subjects but four parallel subtopics of a single theme (the digital divide among older adults in Korea), delivered through an identical prompt template in which only the subtopic phrase was substituted, with a structurally parallel two-part answer (current situation; domestic policy cases) and matched conversational scenario, information density, visual style, and overall length (Section 2.2). Consistent with this control, total fixation time was nearly identical across the four conditions (means 46.6–47.2 s; Section 2.5), indicating that no topic attracted more overall reading than another and that no self-reported measure differed across conditions (Table 8; all means within 4.35–5.39): a sharp topic-interest or difficulty gradient would be expected to leave some trace in these self-reports, yet the large ocular differences were accompanied by comparatively flat subjective profiles, a pattern more readily attributed to the layout manipulation than to content. A residual content contribution nonetheless cannot be excluded because topic was held fixed within each layout rather than counterbalanced across it; the definitive remedy is a fully crossed design that rotates every claim set across every layout, which, together with the order counterbalancing noted below, a single Latin-square assignment would achieve at once. Relatedly, the number of individually visible source items differed across layouts (seven chips; three cards plus a “show all” control; four preview entries beneath a “10 sources” header; section-final link lists), so layout and on-screen source count are partially entangled as well, and the fully crossed follow-up should hold the displayed item count constant.

Third, randomized order was not fully balanced, and presentation position significantly affected all gaze outcomes. The condition effects remained in order-adjusted sensitivity models and in a leave-out reanalysis excluding the 11 participants who saw the panel condition first (Section 3.9), but a Latin-square or completely counterbalanced design is needed in replication.

### 5.3. Ecological Validity and Measurement Scope

Fourth, the stimuli were static screens presented for a fixed 60 s exposure without click interaction. This choice maximized the comparability of cumulative gaze data across participants [49], but it differs from natural use, where users stop when satisfied or open links, and it forecloses observation of the downstream verification act itself: clicking through, reading the source, and returning. Because participants repeatedly cited prior experiences of broken or irrelevant links as a source of distrust, future work should combine gaze tracking with interactive prototypes and click-through logging. All 92 trials did contain at least one source fixation, but the fixed exposure truncates the discovery-latency distribution at 60 s and produced strongly right-skewed latencies (in the hyperlink condition, the TTFF standard deviation exceeded the mean); survival-style models of discovery latency (e.g., Kaplan–Meier curves) would characterize such distributions more naturally in future designs.

Fifth, gaze does not prove comprehension or verification. We did not manipulate source correctness, measure claim–source mapping accuracy, record source opening, test recall, or assess whether trust changed appropriately when a source was strong versus weak. A decisive follow-up should factorially manipulate layout, source-region size, and citation validity (accurate vs. irrelevant vs. fabricated sources) while measuring the full Notice–Attend–Map–Act pathway, testing whether the mapping-rich formats identified here actually improve users’ detection of unfaithful citations rather than merely their comfort. Sixth, interview coding was performed by the first author and reviewed by the corresponding author, without independent dual coding or an inter-rater reliability statistic, and the moderation analyses by usage frequency, platform versatility, and major were exploratory and based on small strata; both should be treated as hypothesis-generating.

### 5.4. Generalizability and Future Directions

Finally, all four answers concerned a single topical domain (the digital divide among older adults) rendered in a single Korean-language chatbot shell on a desktop 60 Hz eye tracker. The pupillometric findings in particular await confirmation with baseline-corrected, luminance-controlled designs on higher-precision, higher-sampling-rate hardware (the 60 Hz stream also bounds event timing to approximately 16.7 ms). Generalization to other domains (e.g., medical or financial queries where perceived risk is higher), other languages, longer multi-turn conversations, free-viewing tasks, and mobile form factors (where a right-hand panel is not even feasible) remains to be established, as does the interplay between content quality and attribution format, including possible compensation effects.

## 6. Conclusions

This study provides, to our knowledge, the first gaze-based comparison of four source-attribution UI typologies abstracted from contemporary AI chatbots that preserves the participant-by-condition experimental unit. Using a within-subjects eye-tracking experiment (*N* = 23; 92 trials) that triangulated ocular metrics, pupillometry, post-stimulus surveys, and post-interviews across four attribution formats (inline component chips, card lists, side panels, and raw hyperlinks), we quantified how format shapes the discovery, scrutiny, and cognitive cost of source cues under a realistic evidence-evaluation task. This constitutes a quasi-experimental mapping of commercial attribution typologies rather than a fully crossed factorial design.

Across these measures, and within this sample of young, AI-experienced university students, the four formats produced a sharply structured attentional landscape: the card list type was discovered almost immediately, whereas raw hyperlinks were found last; the stand-alone chunked formats (the side panel and card list) drew the longest total dwell and the most fixations, while the area-normalized analysis showed that the inline component and card list formats concentrated attention most efficiently within far smaller footprints. Exploratory pupillometry was consistent with elevated processing load for the information-dense side panel alone, with the remaining three layouts being statistically indistinguishable; this physiological result is provisional (Section 3.6). In hypothesis terms, H2 was supported, H4 was supported descriptively, H1 was only partially supported (the card list was detected fastest as predicted, but the side panel was discovered later than the inline component), and H3 was not supported: the familiar hyperlink format neither reduced processing load relative to the other layouts nor earned higher perceived trust. The dissociation at the heart of this study is thus asymmetric in evidential strength: its attentional half is established by large, order-robust effects, whereas its subjective half is an absence of detected differences under single-item measurement, not demonstrated equivalence (Section 3.10). A fully crossed, Latin-square replication that also equates source count and area is the clear next step.

For the design of trustworthy AI systems, the implication we draw is this: citations should earn trust not by being conspicuous but by being verifiable in place. Attribution UIs should provide pre-click identifiability (institution and title, never bare URLs or domain-only chips), sentence-level claim–source mapping (mutual highlighting between answer and evidence), and in situ original-content preview, organized as a two-stage architecture of persistent claim-adjacent cues followed by progressively disclosed detail, so that verification becomes a low-friction default rather than an exhaustive burden. Methodologically, preserving the participant-by-condition unit is essential whenever layouts contain unequal source AOIs. Future research should test whether such verification-affording formats improve users’ actual detection of unfaithful citations across domains, populations, and interactive settings, closing the loop between where users look, what they trust, and what is in fact true.

## Figures and Tables

**Table 1:** Experimental manipulations across Stimuli A–D: placement, pre-click information content, and body-text mapping of source attribution cues. Rows marked “Provided (common)” denote procedures held constant across conditions (controls rather than manipulations).

Property	A (Inline Component)	B (Card List)	C (Side Panel)	D (Raw Hyperlink)
Placement of sources	Inline chip at the end of each supported line (bullet) of the answer	Three source cards plus a “show all” control, arranged horizontally above the answer body	Separate panel to the right of the answer body	Text links listed beneath each answer section
Pre-click information	Short chip showing an abbreviated domain only; no source title or institution visible before clicking	Card showing source title and URL	Preview showing title and body summary of the source	Bare URL text only; no title before clicking
Mapping to body text	1:1 matching at sentence level	No mapping cue (aggregated at top)	No mapping cue (aggregated at right)	1:1 matching at section level
Task interstitial page	Provided (common)	Provided (common)	Provided (common)	Provided (common)
Post-stimulus survey	Provided (common)	Provided (common)	Provided (common)	Provided (common)

**Table 2:** Experimental conditions and source-region geometry.

Cond.	Layout	Visual logic	Source AOIs	Merged Area (px^2^)
A	Inline component	Small claim-adjacent source badges distributed through the answer	7	36,423
B	Card list	Three semantically labelled cards plus a “show all” control, grouped above the answer	4	82,185
C	Side panel	Persistent source summaries in a right-side panel	4	307,441
D	Raw hyperlink	Raw textual links placed near the end of relevant sections	2	97,545

**Table 3:** Participant-level outcome definitions.

Outcome	Operational Definition	Unit	Interpretive Target
TTFF	Start time of the first fixation hitting any source AOI	seconds	Source discoverability
TDF	Sum of durations for all source-hit fixation events	seconds	Total source-directed processing
Fixation-time ratio	Source TDF divided by all fixation duration in the trial	%	Relative attention allocation [33,34]
Fixation count	Number of source-hit fixation events	count	Repeated/sustained processing (cumulative fixation events)
Area density (secondary)	Source TDF (in ms) divided by merged source area ×10,000	ms/10k px^2^	Spatial concentration, not “area-corrected” efficacy [53,65]
Pupil diameter (exploratory)	Mean pupil diameter during whole fixations	mm	Cognitive load

**Table 4:** Survey instruments: operational constructs for evaluating screen readability, information-seeking flow, and UI-attributable trust.

Construct	Description	Scale	No. of Items
Readability	Visual legibility of the screen (font size, line spacing); baseline UI design quality	7-point Likert; open-ended	1
Information-seeking flow	Whether the layout effectively guides the flow of information search	7-point Likert; open-ended	1
Perceived trustworthiness	Impression of trust conveyed by the screen composition itself, holding content constant	7-point Likert; open-ended	1
Behavioural intention indicator	Willingness to use the answer in one’s own assignment or material (behavioural-intention proxy of trust)	7-point Likert; open-ended	1

**Table 5:** Raw-scale participant-level descriptives for the four primary gaze outcomes (*N* = 23 per condition).

Cond.	TTFF M (SD), s	TDF M (SD), s	Ratio M (SD), %	Count M (SD)
A. Inline component	7.34 (7.90)	4.87 (3.46)	10.49 (7.15)	15.26 (7.82)
B. Card list	0.96 (1.63)	9.11 (4.50)	19.54 (9.67)	36.91 (16.88)
C. Side panel	11.17 (15.06)	10.77 (7.60)	22.83 (15.61)	46.39 (29.76)
D. Raw hyperlink	15.65 (16.51)	4.35 (3.67)	9.40 (8.00)	16.39 (13.81)

**Table 6:** Mean time to first fixation (TTFF) and interpretation by source attribution UI type (merged-source AOI, participant level).

UI Type	Mean TTFF	Interpretation
A. Inline component	7.34 s	Intermediate (descriptively; n.s. vs. C and D after Holm); discovered in the course of reading the body text
B. Card list	0.96 s	Fastest; cards captured almost immediately at onset
C. Side panel	11.17 s	Despite its large area, first capture was delayed (descriptively; n.s. after Holm and order-sensitive; see Section 3.9)
D. Raw hyperlink	15.65 s	Slowest descriptively (n.s. vs. A and C after Holm); bare text links show weak initial salience

**Table 7:** Repeated-measures ANOVA on transformed gaze outcomes; Holm p adjusts the four primary omnibus tests as one family. F ratios are computed at the nominal degrees of freedom shown in the column header (3, 66); each pGG evaluates that F at Greenhouse–Geisser-corrected degrees of freedom using the tabulated ε. Logarithms are natural throughout, and TTFF was transformed in seconds. No trial produced a fixation-time ratio at the boundary of 0 or 1, so no boundary adjustment preceded the logit transform.

Outcome	Transform	F(3, 66)	εGG	pGG	Holm p	Partial η^2^
TTFF	ln(1 + x)	11.47	0.759	<0.001	<0.001	0.343
TDF	ln	12.58	0.843	<0.001	<0.001	0.364
Fixation-time ratio	logit	12.61	0.885	<0.001	<0.001	0.364
Fixation count	ln	19.13	0.820	<0.001	<0.001	0.465

**Table 8:** Descriptive statistics of subjective evaluations by UI type (seven-point Likert scale; *N* = 23).

Construct	A (Inline Component)	B (Card List)	C (Side Panel)	D (Raw Hyperlink)
Readability, M (SD)	4.78 (1.68)	5.39 (0.99)	5.22 (1.17)	5.26 (1.10)
Information-seeking flow, M (SD)	5.22 (1.62)	4.96 (1.19)	4.35 (1.50)	5.00 (1.21)
Perceived trustworthiness, M (SD)	5.00 (1.48)	4.65 (1.47)	5.09 (1.47)	4.57 (1.38)
Behavioural intention indicator, M (SD)	5.17 (1.53)	4.87 (1.32)	5.17 (1.50)	4.74 (1.25)

**Table 9:** Improvement demands elicited in post-interviews, by UI type (*n* = number of participants voicing the theme, of 23).

UI Type	Improvement Theme	*n*
A. Inline component	Expose textual identifiers (article title, institution) before click	9
A. Inline component	Reduce the physical size of the chip; refine toward a footnote-like form	5
A. Inline component	Add in-place preview interaction instead of navigating away	2
B. Card list	Provide organic body–card mapping (index numbers, colour linkage, hover highlight)	8
B. Card list	Reposition sources after (not before) the answer content	5
B. Card list	Enrich card content beyond title and link	2
C. Side panel	Mutual highlighting/index matching between body and panel	10
C. Side panel	Relieve visual density; remove duplicated text; adopt collapsible sections	4
C. Side panel	Add a direct-link icon from panel entries to original sources	1
D. Raw hyperlink	Never expose raw URLs; always attach titles and institutional metadata	12
D. Raw hyperlink	Place each source immediately beneath the paragraph it supports	3
D. Raw hyperlink	Provide hover previews (title + summary tooltip) before navigation	2

**Table 10:** Integrated interpretation of quantitative and interview evidence.

**Layout**	**Gaze Signature**	**Reported Strength**	**Reported Barrier**	**Design Implication**
A. Inline component	Moderate discovery; low engagement	Claim adjacency	Source identity hidden pre-click	Keep adjacency; show provenance
B. Card list	Fastest discovery; high engagement	Grouping and readability	Weak claim mapping; pre-emption	Overview plus explicit mapping
C. Side panel	Slow discovery; highest engagement	Rich detail and trust	Dense; costly mapping	Progressive disclosure; bidirectional link
D. Raw hyperlink	Slowest discovery; low engagement	Familiar link convention	Raw URLs lack semantic value	Use titled, attributable links

**Table 11:** Summary of hypothesis testing results.

ID	Hypothesis	Result	Key Evidence
H1	Stand-alone source UIs (card list, panel) will be detected faster than inline UIs (component, hyperlink).	Partially supported	Card list fastest (0.96 s) and hyperlink slowest (15.65 s), as predicted; however, the peripherally placed panel (11.17 s) was discovered later than the inline component (7.34 s), an estimate partly order-inflated (Section 3.9). Omnibus TTFF effect significant (raw F(1.83, 40.2) = 6.40, pGG = 0.005, ηp^2^ = 0.225; ln(1 + x)-transformed F(2.28, 50.1) = 11.47, ηp^2^ = 0.343). Spatial placement within the natural entry path, rather than visual independence per se, appears decisive for initial discovery.
H2	Chunked stand-alone UIs will elicit greater visual attention and longer fixation durations than non-chunked UIs.	Supported	TDF: panel 10.77 s and card 9.11 s vs. component 4.87 s and hyperlink 4.35 s; parallel ordering and significant effects in fixation-time ratio and fixation count (all pGG < 0.001), with cards and panel exceeding chips and links pairwise.
H3	The familiar hyperlink type will induce lower cognitive load (H3a) and higher perceived trustworthiness (H3b).	Not supported (H3a assessed only on an exploratory index)	H3a: pupil diameter, an exploratory index (Section 3.6), did not single out the hyperlink as lowest load: the three non-panel layouts were statistically indistinguishable, and only the side panel showed elevated diameter (Section 3.6). H3b: trustworthiness for the hyperlink (M = 4.57) was descriptively the lowest of the four and did not differ significantly (Section 3.10).
H4	Initial detection speed (TTFF) will be dissociable from verification dwell and trust formation.	Supported (descriptively)	Fast discovery did not entail deep processing or preference: rank orders of TTFF, TDF, and best-UI nominations diverged, and no attentional advantage translated into significant subjective differences. Within-participant correlations corroborate the dissociation (*n* = 92, df = 68): r(TTFF, TDF) = −0.40, *p* < 0.001; r(TTFF, trust) = −0.20, *p* = 0.102; r(TDF, trust) = 0.10, *p* = 0.388 (descriptive, not confirmatory).

**Table 12:** Condition-level summary of gaze, pupil, and preference outcomes by source-attribution layout. Values are raw-scale condition means for the gaze and pupil metrics and nomination counts for the best-UI and worst-UI questions; inferential detail is reported in Table 5, Table 6, Table 7 and Table 8 and Section 3.2, Section 3.3, Section 3.4, Section 3.5, Section 3.6, Section 3.7, Section 3.8, Section 3.9, Section 3.10 and Section 3.11.

Layout	TTFF (s)	TDF (s)	Ratio (%)	Count	Pupil (mm)	Best Votes	Worst Votes
A. Inline component	7.34	4.87	10.49	15.26	3.17	9	6
B. Card list	0.96	9.11	19.54	36.91	3.20	5	5
C. Side panel	11.17	10.77	22.83	46.39	3.30	7	4
D. Raw hyperlink	15.65	4.35	9.40	16.39	3.19	1	8

## Data Availability

The original contributions presented in this study are included in the article. Further inquiries can be directed to the corresponding author.

## Abbreviations

AOI	Area of Interest
TOI	Time of Interest
TTFF	Time to First Fixation
TDF	Total Duration of Fixations
LLM	Large Language Model
RM-ANOVA	Repeated-Measures Analysis of Variance
GG	Greenhouse–Geisser (correction)
UI	User Interface

## Appendix A. Survey and Interview Instruments

**Table A1:** Post-stimulus survey items and post-interview protocol.

Instrument	Item	Scale
Post-stimulus survey (after each of A–D)	Was the overall user interface of the screen you just viewed (font size, line spacing, etc.) generally comfortable to read? [Readability]	7-point Likert; verbal
Post-stimulus survey	Did the composition of the screen help you grasp the key information quickly? [Information-seeking flow]	7-point Likert; verbal
Post-stimulus survey	Did the composition of the screen give the impression that the information was trustworthy? [Perceived trustworthiness]	7-point Likert; verbal
Post-stimulus survey	Did you feel you would want to use the answer on this screen in your own assignment or material? [Behavioural intention indicator]	7-point Likert; verbal
Post-interview	Q1. Did the manner of source attribution influence your trust?	Open-ended
Post-interview	Q2. Which UI was best? Freely describe and sketch improvements.	Open-ended; sketching
Post-interview	Q3. Which UI was worst? Freely describe and sketch improvements.	Open-ended; sketching
Post-interview	Q4. Any improvement ideas for the remaining UIs? Freely describe and sketch.	Open-ended; sketching

## Appendix B. Data Quality and Order Allocation

Presentation-position counts were A/B/C/D = 4/4/11/4 in position 1; 6/8/3/6 in position 2; 7/6/4/6 in position 3; and 6/5/5/7 in position 4. The imbalance is visualized together with the valid-gaze distribution in Figure A1. A leave-out sensitivity reanalysis excluding the 11 participants for whom condition C appeared first is reported in Section 3.9.

## Appendix C. Artifact Analyses for the Exploratory Pupillometry (Section 3.6)

Four artifact accounts were examined. First, mean stimulus luminance was nearly constant across the four screens (245.0–247.8 on a 0–255 scale, a spread of about 1%). Second, because pupil-size estimates from video-based trackers vary with gaze angle (pupil foreshortening; [85]) and gaze in the panel condition was systematically more eccentric (mean fixation x ≈ 1027 px versus 850–911 px in the other conditions, a rightward shift of roughly 3.2–4.9°), the comparison was recomputed using only fixations outside the source regions (i.e., while participants viewed the comparably positioned answer body): the panel’s elevation persisted (M = 3.28 mm vs. 3.17–3.20 mm; F(2.15, 47.3) = 7.83, pGG < 0.001, partial η^2^ = 0.262; vs. A and B, pHolm = 0.011; vs. D, pHolm = 0.064), indicating that gaze eccentricity alone does not explain the effect, although a residual foreshortening contribution cannot be excluded.

Third, baseline-corrected change scores were not computable because the analytic export contains only the stimulus intervals; together with uncontrolled gaze-contingent local luminance and the announced-but-inaccessible source count in the panel header (10 announced, four visible, no scrolling available), which may itself have induced frustration-related load, the load interpretation remains exploratory. Order-linked arousal drift is similarly unlikely to mimic the effect, as presentation order was randomized and position-adjusted models are reported in Section 3.9.

A fourth account is specific to the panel: the side panel condition necessarily reflowed the answer column to a narrower width (Section 2.2), so its longer dwell and larger pupil may partly reflect altered reading load rather than the attribution format alone.

## Appendix D. Exploratory Stratified Observations by User Characteristics (Section 3.7)

Usage frequency. Low-frequency users showed a tendency toward slightly faster TTFF on source AOIs and longer dwell on the component, card list, and hyperlink regions, consistent with an active, wide-ranging search for credibility cues by users less habituated to AI answers. High-frequency users, by contrast, exhibited an efficient scanning profile, briefly confirming the existence of sources on the basis of familiar layout schemata. The exception was the information-dense panel, where they dwelled longer, apparently exploiting it as a context-verification tool.

Platform versatility. Multi-platform users discovered source AOIs faster overall, plausibly reflecting perceptual expertise accumulated across heterogeneous AI interfaces, and dwelled consistently longer than single/dual-platform users on the card list, panel, and hyperlink regions, with the most dramatic gap on the panel (roughly a two-fold difference in mean dwell). The card list type was discovered fastest by both groups, marking it as a universally discoverable format independent of platform experience.

Academic major. Design majors showed relative advantages on the visually polished card list type and the guideline-conforming hyperlink type and maintained longer reading of the section-end link lists, a pattern of adherence to established visual hierarchies and standards. Non-design majors reached the utility-oriented component and panel regions faster and inspected the chunked card and panel structures longer, a pattern of pragmatic, structured cross-checking that treats aggregated source modules as verification tools regardless of aesthetic refinement. These stratified analyses were exploratory and based on small strata; they should be treated as hypothesis-generating (Section 5).
